# Supplementary material for: Implementing a hybrid cognitive-behavioural therapy for pain-related insomnia in primary care: lessons learnt from a mixed-methods feasibility study
Source: BMJ Open. 2020 Mar 18;10(3):e034764. doi: 10.1136/bmjopen-2019-034764 (PMC7150590; doi:10.1136/bmjopen-2019-034764)
Supplement: Author's manuscript [file bmjopen-2019-034764.draft_revisions.pdf]

BMJ Open

BMJ Open is committed to open peer review. As part of this commitment we make the peer review history of every article we publish publicly available.

When an article is published we post the peer reviewers' comments and the authors' responses online. We also post the versions of the paper that were used during peer review. These are the versions that the peer review comments apply to.

The versions of the paper that follow are the versions that were submitted during the peer review process. They are not the versions of record or the final published versions. They should not be cited or distributed as the published version of this manuscript.

BMJ Open is an open access journal and the full, final, typeset and author-corrected version of record of the manuscript is available on our site with no access controls, subscription charges or pay-per-view fees (http://bmjopen.bmj.com).

If you have any questions on BMJ Open's open peer review process please email info.bmjopen@bmj.com

BMJ Open

Implementing a hybrid cognitive-behavioural therapy for pain-related insomnia in primary care: Lessons learned from a mixed-methods feasibility study

Journal:	*BMJ Open*
Manuscript ID	bmjopen-2019-034764
Article Type:	Original research
Date Submitted by the Author:	04-Nov-2019
Complete List of Authors:	Tang, Nicole; University of Warwick, Psychology<br>Moore, Corran; University of Leicester, Psychology<br>Parsons, Helen; University of Warwick, Division of Health Sciences<br>Sandhu, Harbinder; University of Warwick Warwick Medical School, Clinical Trials Unit<br>Patel, Shilpa; Warwick University, Division of Health Sciences<br>Ellard, David; University of Warwick, Clinical Trials Unit;<br>Nichols, Vivien; University of Warwick, Warwick Clinical Trials Unit<br>Madan, Jason; University of Warwick, Warwick Medical School<br>Collard, Victoria; University of Warwick, Psychology<br>Sharma, Uma<br>Underwood, Martin; Warwick University, Warwick Medical School
Keywords:	PAIN MANAGEMENT, SLEEP MEDICINE, Sleep medicine < ANAESTHETICS, MENTAL HEALTH, PRIMARY CARE, QUALITATIVE RESEARCH

SCHOLARONE™
Manuscripts

I, the Submitting Author has the right to grant and does grant on behalf of all authors of the Work (as defined in the below author licence), an exclusive licence and/or a non-exclusive licence for contributions from authors who are: i) UK Crown employees; ii) where BMJ has agreed a CC-BY licence shall apply, and/or iii) in accordance with the terms applicable for US Federal Government officers or employees acting as part of their official duties; on a worldwide, perpetual, irrevocable, royalty-free basis to BMJ Publishing Group Ltd ("BMJ") its licensees and where the relevant Journal is co-owned by BMJ to the co-owners of the Journal, to publish the Work in this journal and any other BMJ products and to exploit all rights, as set out in our licence.

The Submitting Author accepts and understands that any supply made under these terms is made by BMJ to the Submitting Author unless you are acting as an employee on behalf of your employer or a postgraduate student of an affiliated institution which is paying any applicable article publishing charge ("APC") for Open Access articles. Where the Submitting Author wishes to make the Work available on an Open Access basis (and intends to pay the relevant APC), the terms of reuse of such Open Access shall be governed by a Creative Commons licence – details of these licences and which Creative Commons licence will apply to this Work are set out in our licence referred to above.

Other than as permitted in any relevant BMJ Author's Self Archiving Policies, I confirm this Work has not been accepted for publication elsewhere, is not being considered for publication elsewhere and does not duplicate material already published. I confirm all authors consent to publication of this Work and authorise the granting of this licence.

For peer review only

BMJ OPEN

Implementing a hybrid cognitive-behavioural therapy for pain-related insomnia in primary care: Lessons learned from a mixed-methods feasibility study

N. K. Y. Tang[1], C. Moore[1,4], H. Parsons[2, 3], H. Sandhu[2], S. Patel[2], D. R. Ellard[2], V.P. Nichols[2], J. Madan[2], V. E. J. Collard[1], U. Sharma[3], and M. Underwood[2, 5]

[1]Department of Psychology, University of Warwick, Coventry, UK

[2]Clinical Trials Unit, Warwick Medical School, University of Warwick, Coventry, UK

[3]Patient and Public Involvement representative

[4]School of Psychology, University of Leicester, Leicester, UK

[5] University Hospitals of Coventry and Warwickshire

Corresponding Author:

Nicole K. Y. Tang

Department of Psychology, University of Warwick

Gibbet Hill Road, Coventry, CV4 7AL, UK

Phone: +44 (0)2476150556

Email: n.tang@warwick.ac.uk

Keywords: Chronic pain, Insomnia, Sleep, Cognitive Behaviour(al)Therapy (CBT), Psychological Treatment, Hybrid CBT, Patient and Public Involvement (PPI)

Word counts: 5570 (with direct quotes), 4978 (without direct quotes)

No. of box, table, and figure: 1, 3, and 2

For peer review only

Acknowledgments

The authors thank the trial participants and the participating primary care centres for their help in patient recruitment.

Author Contributions

All authors have contributed to the interpretation of the data and manuscript preparation. NKYT was principal investigator and led the project. NKYT, HP, HS, SP, DE, JM US and MU were involved in the design of the study. NKYT, HS, SP led the treatment development and therapist training, with support from US. CM co-ordinated the recruitment of patients and data collection. VPN, DE, and VEJC conducted the qualitative interviews whilst HP conducted the quantitative data analysis.

Funding

This paper presents independent research funded by the NIHR under its Research for Patient Benefit (RfPB) Programme (Grant Reference Number PB-PG-0213-30121). The views expressed are those of the author(s) and not necessarily those of the NHS, the NIHR or the Department of Health and Social Care.

Competing interests

All authors have completed the ICMJE uniform disclosure form. HS & SP are directors of Health Psychology Services Ltd which in part provides psychological treatments for those with chronic pain. HS is chief investigator and co-investigator on other NIHR funded projects. MU was Chair of the NICE accreditation advisory committee until March 2017 for which he received a fee. He is chief investigator or co-investigator on multiple previous and current research grants from the UK National Institute for Health Research, Arthritis Research UK and is a co-investigator on grants funded by the Australian NHMRC. He is an NIHR Senior Investigator. He has received travel expenses for speaking at conferences from the professional organisations hosting the conferences. He is a director and shareholder of Clinvivo Ltd that provides electronic data collection for health services research. He is part of an academic partnership with Serco Ltd related to return to work initiatives. He is a co-investigator on a study receiving support in

For peer review only - http://bmjopen.bmj.com/site/about/guidelines.xhtml

kind from Stryker Ltd. He has accepted honoraria for teaching/lecturing from CARTA & Sterling Events. He is an editor of the NIHR journal series, and a member of the NIHR Journal Editors Group, for which he receives a fee. NKYT received grant funding as co-investigator from NIHR for other projects and current funding from the Medical Research Council as principal investigator. NKYT is also a member of the trial steering committee of an ongoing trial funded by the NIHR.

Ethics approval

The National Health Service (NHS) ethical approval was given by the National Research Ethics Service (NRES) Committee West Midlands – Solihull, REC Number: 14/WM/1053. All participants gave informed consent before commencing the study.

Data sharing statement

No additional data are available.

Open Access

For peer review only

Abstract

Objectives: To test the feasibility of implementing a brief but intensive hybrid cognitive behaviour therapy (Hybrid CBT) for pain-related insomnia

Design: Mixed-methods, with qualitative process evaluation on a two-arm randomised controlled feasibility trial

Setting: Primary care

Participants: Twenty-five adult patients with chronic pain and insomnia

Intervention: Hybrid CBT or Self-help control intervention

Primary and secondary outcome measures: Primary outcomes measures were the Insomnia Severity Index and interference scale of the Brief Pain Inventory (BPI). Secondary outcomes measures were the present pain intensity rating from the BPI, Multidimensional Fatigue Inventory, Hospital Anxiety and Depression Scale, and EuroQol.

Results: Fourteen participants were randomised to receive Hybrid CBT, 11 to receive the self-help control treatment. Of the 14 in the Hybrid CBT group, 9 (64%) completed all four treatment sessions (4 discontinued due to poor health; 1 due to time constraints). The total number of participants completing the 12-week and 24-week follow-ups were 12 (6 in each group; Hybrid CBT: 43%; Self-Help: 55%) and 10 (5 in each group; Hybrid CBT: 36%; Self-help: 45%). Based on the data available, candidate outcome measures appeared to be sensitive to changes associated with interventions. Thematic analysis of pre-post intervention interview data revealed satisfaction with treatment content among those who completed the Hybrid CBT whereas those in the Self-help control treatment wanted more contact hours and therapist guidance. Other practical suggestions for improvement included shortening the duration of each treatment session, reducing the amount of assessment paperwork, and minimising the burden of sleep and pain monitoring.

Conclusion: Important lessons were learnt with regards to the infrastructure required to achieve better patient adherence and retention. Based on the qualitative feedback provided by a subset of treatment completers, future trials should also consider lowering the intensity of treatment and streamlining the data collection procedure.

Trial registration: ISRCTN 17294365

For peer review only - http://bmjopen.bmj.com/site/about/guidelines.xhtml

Strengths and limitations of this study

- First feasibility trial in the UK to evaluate a new, brief but intensive Hybrid CBT for pain-related insomnia compared to self-help control in primary care.
- The Hybrid CBT was manualised, the delivery of which was supported by a comprehensive therapist training programme.
- Patient recruitment was tested in three different health centres of different demographic compositions and different socio-economic backgrounds.
- The mixed-methods approach provided both quantitative and qualitative information to inform the design and planning of a definitive trial.
- Rates of attrition and loss to follow-up were high in both arms.

CONSORT Checklist attached

CORE-Q Checklist attached

For peer review only

Introduction

Chronic pain is a major burden to primary care, accounting for five million GP appointments each year in the UK (1, 2). These pain patients usually present with multiple symptoms, with insomnia being one of the most common and disruptive comorbidities (2-4). In hospital pain clinics, as many as 90% of the patients report insomnia of a severity that warrants clinical attention (3, 5-8).

Conventionally sleep disturbance is seen as a secondary symptom to pain, but recent research has shown that poor sleep is actually a key driver for persistent pain and its associated distress and disability (9-13). Additionally, untreated insomnia is a significant risk factor for adverse health outcomes, e.g., hypertension, cardiovascular disease, obesity, diabetes, respiratory diseases and even increased mortality(14-20).

Although better sleep has long been emphasised by pain patients as an important treatment outcome (21, 22), insomnia is rarely a focus in pain management programmes. In primary care where most chronic pain patients are managed, hypnotics continue to be first-line treatments for insomnia despite the limited evidence supporting their long-term efficacy and safety (23). Their prolonged use can result in undesirable side effects, increasing the risks of falls, road traffic accidents, dementia and mortality in the long term, particularly in older adults (24-28). The risks multiply when the effect of polypharmacy is factored in. The combined use of benzodiazepines and opioids produce significant respiratory depression and is thought to contribute to the recent sharp rise in unintentional prescription drug overdose deaths (29).

Psychological interventions offer a promising treatment alternative. The efficacy of cognitive behaviour therapy for primary insomnia (CBT-I) has been demonstrated by multiple systematic reviews and meta-analyses (30-36). However, these treatments are often not available for chronic pain patients because of the lack of an empirically-validated treatment protocol adapted and tailored for this population (37), a shortage of skilled therapists (38-40), and an absence of essential infrastructure for CBT delivery in primary care (28, 30, 41).

For peer review only - http://bmjopen.bmj.com/site/about/guidelines.xhtml

We have recently evaluated a talking therapy specifically modified for patients with pain-related insomnia (42). The intervention simultaneously tackled chronic pain and insomnia, combining select components of CBT-I with interventions targeting the cognitive-behavioural processes maintaining chronic pain. The Hybrid CBT was delivered as an individual therapy over four weeks through weekly two-hour sessions. The treatment dosage was eight hours in total, approximating the optimal dose recommended for CBT for insomnia disorders (43) within a stepped care model (39). In our pilot study with patients recruited from hospital pain clinics (i.e., secondary care), the Hybrid CBT was associated with greater improvement in sleep at post-treatment compared with a symptom-monitoring control procedure. Pain intensity did not change, but the Hybrid CBT was associated with greater reductions in pain interference, fatigue and depression than control (42).

The current study tested the feasibility of adapting and implementing the Hybrid CBT in primary care, using a mixed-methods approach. With a small patient sample across three primary care centres from localities with different social-economic and demographic characteristics, our overarching aim was to generate information to inform the development of a definitive randomised controlled trial (RCT) for evaluating the clinical outcomes and cost-effectiveness of the Hybrid CBT within the UK National Health Service (NHS) setting. The focus of the current study was therefore not on detecting differences in outcomes between the Hybrid CBT and the control groups, but on evaluating the technical and logistic feasibility of a full-scale study. Hence, in this article, we report the methods and findings from the feasibility trial, along with qualitative findings based on our process evaluation of patient experience.

Methods

Patient and public involvement (PPI)

Two patient representatives with prior training for research involvement were recruited for this study through the Warwick Universities/User Teaching and Research Action Partnership. Our PPI representatives were co-applicants of the grant application and members of the project management committee. They were

involved in most aspects of the study including trial design, therapist training, trial implementation and results discussion. Additionally, a member of our research team with a chronic pain condition gave significant insights into the running of the trial on top of their technical research expertise.

Trial setting and design

The feasibility study was a randomised controlled trial with a multi-centre, parallel-group design situated within primary care. Treatments were offered to adults living with chronic pain and insomnia in the community and delivered by trained health psychologists at the primary care centre from which the patients were recruited. (ISRCTN registration number: 17294365)

Participants were randomly assigned to receive one of two trial treatments for pain-related insomnia, in addition to treatment as usual (TAU). Here, TAU referred to the existing advice and prescribed medications for pain and insomnia that the participants were receiving. The assumption of TAU reflected the clinical reality that most patients with these chronic conditions would have already received some medical advice or treatment for their symptoms.

Treatments

Hybrid CBT

The Hybrid CBT comprised select components of CBT-I and interventions designed to target cognitive-behavioural processes maintaining chronic pain. The core components of the treatment were described in Tang et al. (42) and included sleep psychoeducation, stimulus control therapy, sleep restriction therapy and cognitive therapy for addressing insomnia-related cognitions and behaviours common amongst chronic pain patients. It also included individual formulation, goal setting and behavioural activation, components for reducing pain catastrophising and safety-seeking behavior, and reversing mental defeat for the management of chronic pain. The treatment was manualised for this study to facilitate therapist training; the guiding treatment principles were laid out in the treatment manual to support flexible treatment delivery for this patient group with complex needs.

For peer review only - http://bmjopen.bmj.com/site/about/guidelines.xhtml

The format of the treatment can be described as 'brief, intensive, and concentrated' (44). Each patient allocated to the Hybrid CBT group was offered a total of four individual sessions on a weekly basis. Each session was approximately two hours long. The idea was to maintain the level of treatment content whilst minimising the burden of travel and duration of treatment, which may hinder treatment engagement in this patient group (45).

Self-help control treatment

Existing reading materials were amalgamated (with minimal modification) into four booklets to provide a self-help treatment on managing chronic pain and insomnia (46). The self-help booklets were posted to the patients' homes, one at a time on a weekly basis. The content of the self-help gave equal coverage on chronic pain and insomnia management, providing a good comparison with current available treatment.

Therapists

In the UK, clinical psychologists are not usually a part of the primary care medical team. Patients are often referred to see a psychologist for psychological interventions on an as-needed basis. Other provisions of care exist, e.g., in-house counsellors and local Improving Access to Psychological Therapy (IAPT) teams, but availability of these services varies depending on locality and resource allocation. Previous trials of CBT-I in primary care have recruited nurse/health visitors for the delivery of treatment (47). Given the content (i.e., treatment of two complex health conditions), approach (non-protocol-based CBT), format (individual, brief but intensive) and focus (behaviour change) of the Hybrid CBT, health psychologists were chosen as therapists for this trial.

Following targeted recruitment via health psychology training centres and professional networks across the UK, six health psychologists - fully qualified or in the latter stages of their stage II doctorate – were selected to receive three days of intensive training offered by the team. Three withdrew before the trial commenced due to clashes with existing employment/study commitments (e.g., maintaining private practice and completing other training) and the distance of

For peer review only - http://bmjopen.bmj.com/site/about/guidelines.xhtml

travelling involved in the process of treatment delivery (e.g., from London or Staffordshire, where the recruited therapists were based, to Coventry, Rugby and Warwickshire, where the primary care centres were located). The remaining three (100% female) went on to become the trial's therapists, which involved further training at their own pace via learning resources posted online, case piloting, regular individual supervision by experienced health psychologists on the team (HS, SP, NKYT), and travelling across sites to offer treatment to the patients in their localities.

Patients

We recruited 25 people living with chronic pain, between April 2016 and April 2017 from three primary care centres in Coventry/Warwickshire of different demographic compositions (respectively having 2.1%, 3.7% and 25.8% non-white population) and different social-economic backgrounds (respectively scoring 1, 5 and 8 on the 1-10 Index of Multiple Deprivation) (48).

We identified participants for the study from the electronic registers held by the participating centres. Patients were initially screened by searching each centre's electronic patient records for inclusion criteria. We did two searches, with search terms broadened to include specific medications for the second search.

Inclusion criteria were individuals (i) aged 18 years or above, (ii) English-speaking, (iii) registered with one of the participating centres, (iv) with a history of chronic pain and insomnia (as indicated by their medical records), with (v) pain of at least moderate severity ($\geq$4/10 on a present pain intensity numerical rating scale for at least 6 months), and (vi) clinical insomnia ($\geq$15 on the Insomnia Severity Index, $\geq$3 nights a week, $\geq$1 month in duration). Criteria vi mapped onto the American Academy of Sleep Medicine Research Diagnostic Criteria for Insomnia Disorder (49), which is consistent with the DSM-5 diagnostic criteria although the latter adopted a three-month duration criteria (50).

Participants taking pain/sleep medications on a stable regimen were included if they met criteria of the study. However, we excluded potential participants with

For peer review only

diagnosed or suspected medical/psychiatric/sleep disorders for which CBT-I was contraindicated as first-line treatment or those who had recently enrolled in or were completing a pain management programme, or other psychological treatments for pain or sleep.

As this was a feasibility study, no formal power calculation to test the effectiveness of the intervention was possible. The current sample size was determined by practicalities and considerations that it was sufficiently large to ensure that randomisation was acceptable and to buffer against atypical attrition.

A randomisation list was created by the trial statistician (HP) using random blocks of varying sizes (block length=4 or 6). Blocks were generated in groups of patients at a 1:1 ratio, and stratified by centre from which the participants were recruited. Patients were randomised sequentially as they became eligible for inclusion in the study. Allocation was concealed using 'e-envelopes', which were macro-enabled MS Excel files preserved as read-only with automatic saving on any alteration to the file to give an audit log. This method was considered cost-efficient for a small-scale feasibility study. Furthermore, the study statistician only released these e-envelopes and their passwords in small batches on request by the trial coordinator (CM). The study statistician had no contact with participants at any point in the study.

Quantitative outcome measures

We piloted the use of five validated questionnaires to collect data in this population. Our candidate primary outcomes were the Insomnia Severity Index (ISI) (51) and Brief Pain Inventory (BPI) pain interference subscale (52). Candidate secondary outcomes included the present pain intensity rating from the BPI (52), Multidimensional Fatigue Inventory (MFI) general fatigue score (53), Hospital Anxiety and Depression Scale (HAD) (54) and EuroQoL (EQ-5D-5L) (55), for which health utilities were calculated using the UK tariff of the EQ-5D-5L value set (56). The health thermometer score was also reported.

For peer review only - http://bmjopen.bmj.com/site/about/guidelines.xhtml

Several process measures were included as part of the assessment to inform treatment and elucidate the role of hypothesised treatment mechanisms. These were the Pain Catastrophizing Scale (57), Pain Self-Perception Scale (58) Anxiety and Preoccupation about Sleep Questionnaire (59), Dysfunctional Belief and Attitude about Sleep Scale–16 (60), and Pain-specific Dysfunctional Belief and Attitude about Sleep Scale (61).

In addition, the participants were asked to complete a daily sleep diary modified from the Consensus Sleep Diary (62) and to wear an actigraph (Model: MW8, supplied by CamNTech) for a week for baseline and 12-week follow up assessments to examine the feasibility of incorporating objective sleep measures for future trials.

Statistical analysis

As the aim of the current trial was to evaluate study feasibility, not treatment efficacy, no formal between-group analyses were planned. However, planned analyses consisted of the generation of descriptive statistics for all time points and the Cronbach's alpha of the candidate primary outcome measures to assess if internal consistency of these measures was adequate when administered in a chronic pain patient sample with a minor modification (i.e., the sleep item was removed from the BPI to avoid criterion contamination). The candidate primary outcome measures were also checked for correlation to establish if co-primary outcomes were needed for a definitive study.

Qualitative participant interviews

Face-to-face semi-structured interviews were conducted before and after the intervention to explore participants' expectations of the intervention and their overall experience post-intervention. These interviews were carried out individually with a subgroup of participants enrolled in the trial by a Research Fellow who specialises in qualitative health service research but was not involved in the treatment design and delivery process (VPN). The interviewer had no prior contact with the interviewees. Seed questions used to prompt these conversations are shown in Box 1. Timelines of the participant's symptom and treatment journey

For peer review only - http://bmjopen.bmj.com/site/about/guidelines.xhtml

were drawn during the interview and field notes were written immediately post interview to promote reflexivity. The interviews were held at the participant's primary care centre, and lasted for about 60 minutes each. Interviews were audio-recorded on an encrypted digital device. Recordings were transcribed verbatim and anonymised. Data transcription was supported by another doctoral-level Research Fellow with experience in qualitative health care research but again not involved in the study design and treatment delivery process (VEJC). NVivo software was used for managing the data for analysis of the interview transcripts.

Thematic analysis was performed on the transcripts by the CI (NKYT) following the six key processes recommended by Braun and Clarke (63); familiarisation with the data set, generating initial codes, searching for themes, reviewing themes, defining and naming themes, and report production. Codes and themes extracted were then reviewed by the interviewer (VPN) and a second experienced qualitative researcher on the team (DE) to check for accuracy. Comments received were then used to revise the analysis. Themes were extracted with the awareness that questions asked in the pre-intervention interviews were different from the post-intervention interview, and hence the analysis avoided referring to any within-participant change across these interviews. Constant comparisons were applied when analysing the post-intervention interviews, contrasting the experience of the participants assigned to receive Hybrid CBT with those assigned to the Self-help control treatment.

Results

Search results

The searches identified a total of 1,434 potentially eligible patients across the three primary care centres (9.8% of all records). After receiving an invitation to become part of the study, 85 patients responded and were invited to telephone (n=45) and then in-person (n=40) screening to be further assessed for eligibility. Of the 25 (55.6% of screened) participants who were found to be eligible, all were recruited into the study. This corresponded to a pick-up rate of 1.7 per 1000 registered patients and a successful recruitment rate of 1.9 participants per

For peer review only - http://bmjopen.bmj.com/site/about/guidelines.xhtml

month, or 0.6 participants per centre per month. Figure 1 specified the number and reasons for exclusion at screening and subsequent stages.

Baseline participant characteristics

Table 1 summarises the socio-demographic details provided by the study participants at baseline (n=25). At the group level, 56% of the participants were female with an average age of 49 years and a mean BMI of 29. The majority of the participants were white (96%) and married or living as married (60%). Forty-four percent of the participants had secondary level education as their highest educational qualification. At the time of the study, 40% of the participants were in paid work whilst 60% were unemployed, retired or engaged in other forms of activity, with 36% receiving some form of social benefits.

The mean reported duration of pain was 11 years. Most of the participants described their pain as "constant" and the median number of pain sites reported was 5, with lower back as the most commonly identified pain site, followed by neck, shoulders, joints, legs, knees, arms, upper back, head and abdomen. The baseline present pain intensity VAS was 6.1, pain interference score 6.6, and ISI 18.9.

Treatment adherence and attrition

All 14 participants assigned to the Hybrid CBT group completed session 1, nine completed (64.3%) sessions 2, 3, and 4. Of the five participants who discontinued, four gave 'poor' health and one gave 'lack of time' as reasons for withdrawal (Figure 1). Those withdrawn were recruited from primary care centres with more severe deprivation indices.

For the self-help control group, all leaflets were mailed to the participants weekly, as per protocol. No further adherence data were collected.

At 12-week, six participants were successfully followed up in each arm; at 24-week five. This gave an overall participant retention rate of 48% at 12-week and 36% at 24-week.

For peer review only - http://bmjopen.bmj.com/site/about/guidelines.xhtml

Outcome measures

Both primary outcome measures appeared to have excellent internal consistency. The Cronbach's alpha for the cohort was 0.94 (95% CI 0.92-0.97) for the ISI and 0.88 (95% CI 0.87-0.95) for the pain interference score. Even though the BPI| sleep item was dropped to avoid criterion contamination, there was a strong non-zero correlation between ISI and BPI interference score (r = 0.81, 95% CI 0.67-0.89).

Descriptive statistics of all primary and secondary outcome measures, as well as process measures, by assessment time points, are shown in Table 2. No adverse events were reported in either allocation groups.

Participant Interviews

Five patients were interviewed; three were from the Hybrid CBT group and two were from the Self-help control group. All completed both pre- and post-intervention interviews, except one in the Hybrid CBT group who only completed the pre-intervention interview.

Pre-intervention

Discussions in the pre-intervention interviews gave rise to six interesting themes, capturing several prominent psychosocial characteristics of the participants enrolled in the feasibility study. These themes (summarised in Table 3 with illustrative quotes) were concerned with the participants' sense of identity, personal adversities, treatment experience and coping strategies, perceived pain-sleep relationship, satisfaction with current service, and treatment expectations. These themes contextualised the feasibility study, offering finer insights into the life circumstances of those who actually signed up for pain-related insomnia treatment.

Post-intervention

Discussions in post-intervention interviews revealed aspects of the intervention liked and disliked by the two interviewees (as graphically summarised for the Hybrid CBT in Figure 2). Analysis of these factors was carried out separately for the Hybrid CBT and the Self-help control groups, to generate clear suggestions as

For peer review only - http://bmjopen.bmj.com/site/about/guidelines.xhtml

to how each of these interventions could be tweaked and improved from the patient experience perspective.

Hybrid CBT

Besides "very good practical advice" on sleep, both interviewees who completed the Hybrid CBT appeared to most appreciate the intervention for giving them a new understanding of sleep as well as themselves. In particular, they seemed to like the fact that these new insights enabled them to improve their sleep patterns and to change the way they think about and react to situations in life. Their success in improving sleep also appeared to have boosted their confidence in initiating changes in other areas of their lives; a spill-over therapeutic effect.

> *"... You know, understanding how you sleep really... a lot better frame of mind now and a lot better myself you know what I mean? ...Because they're slowly dispersing these worries are, and things like that, a lot to look forward to, and different things, more positive now... [It]changes the way you think a little bit and me talking to [the therapist] that I want to walk more and do more... It makes you think like... I can do something about it, I've done this, I've done um this sleep pattern and that..." (Patient A)*

> *"I'm remembering everything and I really liked the study 'cause it helps me do things different... it's all about retrain(ing) your brain to you know understand our sleep patterns and things we do in our life, what we do and it works it really really works..." (Patient C)*

Both participants interviewed were also very positive about the one-to-one, face-to-face interaction they had with the therapists. They felt that they were being listened to, and really appreciated the fact that the therapists went to the community to offer the intervention in their locality. They also felt that they managed to derive a greater understanding of the treatment materials because they could talk to someone and ask questions as they arose, compared to reading the information on their own. Further, they felt that being able to talk confidentially to someone knowledgeable about their symptoms and experience had allowed them to process difficult emotions that may or may not be directly linked to the experience chronic pain.

For peer review only - http://bmjopen.bmj.com/site/about/guidelines.xhtml

"…the one-to-one sessions are good... They are really good. Um I don't know how it would work in a group." (Patient C)

"…I think you understand it a bit more by talking [to] somebody" (Patient A)

"… I'd go home have a good read about [the treatment materials], some of it I didn't understand but then when I come back I'd ask… or she'd explain to me… write it down and show me that this is the way to do it... So I was learning a new skill… I don't think I'd have took it so serious ... I don't think I would've, just read a couple of leaflets and oh yeah and then popped it down the side, I wouldn't have thought about it…" (Patient C)

"And at least they could come to me and I didn't have to get there. I wouldn't have done that you see?... Cause it's on your door step it's a lot easier ... and you fit it in" (Patient A)

Three issues of the Hybrid CBT required attention. First, although sleep monitoring was an essential component of the intervention, the participants interviewed found completing the sleep diary tedious and possibly sleep-interfering as they felt obliged to clock their activities and remember everything they had done. Second, the participants found components of the sleep restriction and stimulus control therapy hard to follow. Although their sleep became more consolidated as a result of these treatment components, they did not particularly enjoy the experience and felt it was important to be able to personalise the intervention. Third, the participants struggled to apply the sleep restriction therapy during pain flare ups. Further adjustments to the pace and method of the therapy may be required considering that an increase in pain is a possible side effect during the initial stage of the sleep restriction therapy. Application of digital technology may also help reduce the burden of data collection.

"[The monitoring/diary] was a bit monotonous, but then they're not going to know unless you write it down…" (Patient A)

"I didn't like that side of it (the sleep restriction and stimulus control therapy) 'cause I do like to stay in bed…" (Patient C)

"…but then you can have a flare up and then you're struggling…you know?" (Patient A)

"… I was following it all um you know we've come to like an agreement on timing to go to sleep and timing to get up…, but the problem is I've got a bad neck"…"It's really sore and moving it sometimes I can't move it ... Can't go out,

For peer review only - http://bmjopen.bmj.com/site/about/guidelines.xhtml

can't drive, can't do anything cause my necks playing me up, so it knocked my sleep pattern a bit out of proportion…" (Patient C)

Self-help control

The Self-help control intervention was chosen to represent what was the best treatment option available for patients with chronic pain and insomnia in primary care. However, few positives were said about the Self-help control intervention, except that the advice given was "sound" and "clear". Whilst both participants interviewed felt that overall the intervention was helpful, they felt that they were not learning much new information and could not pinpoint any specifics as to why and how the intervention was helpful. Their memories of what was being discussed in the self-help booklets were also very vague. They felt that the information provided in the booklets overlapped with information available in existing self-help books or internet sources. They also indicated that they wanted more contacts with health care professionals rather than being left to their own devices.

"Yeah the study itself is alright…"… "On the sleep [side], the things it gives are clear and can be helpful, but to me I already practiced those things anyway… but I've already read up on lots of things and done, so there wasn't a lot there that was explaining that I could take away and thought I've absorbed something new."" (Patient E)

"So something's obviously helped along the way but I can't put my finger on one exact thing." "[Interviewer: is there anything, is there anyway we could improve it do you think?] In a way perhaps a bit more contact because obviously you have the initial study ... I saw you for that interview sort of in the middle ... And then now, but other than the booklets then you are just sort of left to it.... There's no contact whatsoever.... So some contact would've been useful." (Patient B)

Other feedback from participants assigned to both groups were concerned with the randomisation procedure, intervention format, and data collection method. On randomisation, the participants in the Self-help control group indicated that it "would have been nice to have a choice" whereas those in the Hybrid CBT group imagined that they "wouldn't have bothered" with the treatment or would have benefited less had they been assigned to the Self-help control group. There was

For peer review only

also an agreement from participants of both groups on the intensity of the treatment, with too much to read and/or report. Whilst they did not have an issue with automated data collection devices (i.e., actigraphy), they had found it difficult to complete the sleep diary and recommended that the paper work of the treatment/study to be reduced.

Discussion

Delivering a brief but intensive intervention for the self-management of chronic pain and insomnia in primary care has proved to be challenging. Important lessons were learnt with regard to the infrastructure and trial design required to achieve better patient recruitment, treatment delivery, intervention adherence, and patient retention.

Were the patient identification and recruitment strategies viable?

Two searches were conducted to identify potential participants from electronic records. The initial search that used diagnoses as search terms identified only 263 potential participants across the three participating centres despite the high prevalence rates of both health conditions (64-67). The unusually low return might be explained by the fact that physician records did not always list sleep or chronic pain as diagnoses. A second search using a broadened set of search terms (including medication prescribed) was more successful, identifying 1,434 potential participants (inclusive of the 263 previously identified). Of those who responded to the invitations and were invited to full screening; nearly a third were randomised into the study (63%). Of those who were not randomised, approximately equal numbers were ineligible and declined to attend full screening. This indicates that once found, recruiting patients into the study may not be an issue for a larger study, but capturing interested patients in the first place would be a challenge. Whilst the current pick-up rate of 1.7 per 1000 patients was satisfactory, future trials should multiply the number of recruiting primary care centres to ensure that the recruitment target will be reached in a timely fashion.

For peer review only - http://bmjopen.bmj.com/site/about/guidelines.xhtml

Previous research has found that patients with insomnia tend to trivialise their symptoms and did not seek treatment due to beliefs that one should be able to cope with insomnia alone (68). Despite the brief but intensive nature of the Hybrid CBT, four weekly sessions plus homework and data collection is a significant time commitment. Future trials should consider addressing these unhelpful beliefs during patient recruitment and incorporate an incentive system to motivate eligible participants to commit themselves to treatment. For example, a US trial of a six weekly 90-minutes group CBT for insomnia and pain among older adults with osteoarthritis offered a USD$2 cash incentive in the initial postal invitation (69). Participants in this study were paid volunteers. They received a USD$50 incentive payment after completing the baseline assessment and attending the first class (69). Similarly, an ongoing nurse-led brief insomnia treatment trial in the UK reimburses all participants after each completed follow-up visit; £5 at baseline, £10 at 3-month, £15 at 6-month, and £10 at 12-month (70). The current study offered the treatment for free but did not have the budget to incentivise enrolment and treatment attendance.

What were the rates of attrition?

Loss to follow up was high in this feasibility study, but return rates were generally higher in the Hybrid CBT group than the Self-help control group. Adherence to the Hybrid CBT appeared to be most vulnerable after session 1. Ill health was cited as the main reason for drop-out and incidentally, all patients withdrawn were recruited from centres with more severe deprivation indices. Those who managed to return to session 2 fully adhered to the rest of the treatment programme and appeared to report sizeable improvements across outcome measures. One size does not fit all; it would be important for future trials to identify demographic, socio-economic, and clinical factors that predict treatment suitability, directly answering the ‘what works for whom’ question.

In addition to a dedicated budget for incentivising follow-up rates, practical support (e.g., travel reimbursement, appointment reminders, between-session technical support) may also be required to remove participation barriers and reduce attrition in future trials. Qualitative feedback from our participant

For peer review only

interviews also indicates that streamlining the data collection with the help of digital technologies or the use of a more active control intervention with more patient contact may help buffer against attrition.

How satisfactory and acceptable were the outcome measures and data collection methods?

Candidate outcome measures tested in the current trial appear to be psychometrically sound and have good face validity for the stated purpose of assessment. Both primary and secondary outcome measures showed changes in the direction anticipated for both arms of the trial over time. Care must, however, be taken when interpreting the positive responses that were reported due to biases associated with selective uptake and study attrition. We note that participants who remained in the study had lower levels of pain intensity at baseline than those who withdrew or were lost at the 12-week follow up (BPI severity: 5.9 v 6.4, not tested), although levels of pain interference and insomnia were more similar (BPI interference: 6.1 v 6.2, ISI: 20.3 v 20.1, not tested).

The pattern of change in the outcome measures was approximated by those of the process measures that assessed the hypothesised maintaining factors of pain and insomnia targeted by the treatments. Future trials should consider conducting appropriately designed analyses to examine whether changes in these processes mediate treatment outcomes. The combined use of a sleep diary and actigraphy is an important part of the assessment and treatment process (not reported in detail here), but the implementation of this recommended monitoring procedure proved to be challenging for the participants of the current study. Potential solutions to improve monitoring adherence in future trials may involve reducing the length of the monitoring procedure, more personalised data collection training and support, and the installation of analysis software in participating centres to minimise human errors in data transfer.

Strengths and limitations of this study

This was a small-scale feasibility study with a subgroup of participants being interviewed pre-post treatment for their experience participating in the trial. Findings must be interpreted within its bounds of generalisability.

For peer review only - http://bmjopen.bmj.com/site/about/guidelines.xhtml

For peer review only

Therapist contact and the treatment focus on understanding how sleep works were highly valued by our interviewees. The delivery of the Hybrid CBT at the patient's doorstep was also positively received. Future trials should seek to maintain the quality of contact, although this will have to be supported by a robust health economic analysis that examines the cost-effectiveness of providing the Hybrid CBT in its current format as compared to in group settings or in the form of telemedicine or internet-based intervention.

Whilst no adverse event was formally reported, qualitative feedback suggested that increased pain – especially in the initial treatment period - may be a possible side effect of the sleep restriction component of the treatment. Further investigations into the frequency, timing, and severity of this potential side effect are required to ensure patient safety and, potentially, promote treatment adherence.

A previous meta-analysis has found the short-medium term outcomes of patients with comorbid or primary insomnia receiving bibliotherapeutic self-help were superior to those of waitlist control (71). Although self-help treatment is the best available non-pharmacological treatment options in many primary care settings, the trial team should consider whether it is an acceptable control intervention to offer in future trials. The post-treatment interviews revealed that our participants were not naive to self-help and appeared to be demoralised by the lack of therapist contact and fresh treatment content.

Conclusion

The Hybrid CBT has the potential to fill an unmet clinical need. Through our feasibility trial, a treatment protocol and a corresponding therapist training programme have been developed to make the delivery of this brief but intensive intervention in primary care possible. In its current form, the Hybrid CBT may work for subgroups of individuals who manage to adhere to the programme. Future trials could overcome the challenges highlighted in this feasibility study by broadening recruitment catchment, incorporating an incentive system to motivate treatment uptake, streamlining the treatment to make it even more

For peer review only - http://bmjopen.bmj.com/site/about/guidelines.xhtml

primary care friendly, and simplifying the data collection procedure to make it easier for the patients to take part and provide data for evaluation.

For peer review only

For peer review only - http://bmjopen.bmj.com/site/about/guidelines.xhtml

References

1. Belsey J. Primary care workload in the management of chronic pain. A retrospective cohort study using a GP database to identify resource implications for UK primary care. Journal of Medical Economics. 2002;5(1-4):39-50.
2. Ohayon MM. Relationship between chronic painful physical condition and insomnia. Journal of psychiatric research. 2005;39(2):151-9.
3. Tang NK, Wright KJ, Salkovskis PM. Prevalence and correlates of clinical insomnia co - occurring with chronic back pain. Journal of sleep research. 2007;16(1):85-95.
4. Taylor DJ, Mallory LJ, Lichstein KL, Durrence HH, Riedel BW, Bush AJ. Comorbidity of chronic insomnia with medical problems. Sleep. 2007;30(2):213-8.
5. Bigatti SM, Hernandez AM, Cronan TA, Rand KL. Sleep disturbances in fibromyalgia syndrome: relationship to pain and depression. Arthritis Care & Research: Official Journal of the American College of Rheumatology. 2008;59(7):961-7.
6. Atkinson J, Ancoli-Israel S, Slater MA, Garfin SR, Gillin C. Subjective sleep disturbance in chronic back pain. The Clinical Journal of Pain. 1988;4(4):225-32.
7. Moldofsky H, Scarisbrick P. Induction of neurasthenic musculoskeletal pain syndrome by selective sleep stage deprivation. Psychosomatic medicine. 1976.
8. Wolfe F, Michaud K, Li T. Sleep disturbance in patients with rheumatoid arthritis: evaluation by medical outcomes study and visual analog sleep scales. The Journal of Rheumatology. 2006;33(10):1942-51.
9. Haack M, Mullington JM. Sustained sleep restriction reduces emotional and physical well-being. Pain. 2005;119(1-3):56-64.
10. Smith MT, Edwards RR, McCann UD, Haythornthwaite JA. The effects of sleep deprivation on pain inhibition and spontaneous pain in women. Sleep. 2007;30(4):494-505.
11. Kundermann B, Krieg J-C, Schreiber W, Lautenbacher S. The effects of sleep deprivation on pain. Pain Research and Management. 2004;9(1):25-32.
12. Lentz MJ, Landis CA, Rothermel J, Shaver JL. Effects of selective slow wave sleep disruption on musculoskeletal pain and fatigue in middle aged women. The Journal of rheumatology. 1999;26(7):1586-92.
13. Tang NK, Goodchild CE, Sanborn AN, Howard J, Salkovskis PM. Deciphering the temporal link between pain and sleep in a heterogeneous chronic pain patient sample: a multilevel daily process study. Sleep. 2012;35(5):675-87.
14. Cappuccio FP, Taggart FM, Kandala N-B, Currie A, Peile E, Stranges S, et al. Meta-analysis of short sleep duration and obesity in children and adults. Sleep. 2008;31(5):619-26.
15. Cappuccio FP, D'Elia L, Strazzullo P, Miller MA. Quantity and quality of sleep and incidence of type 2 diabetes: a systematic review and meta-analysis. Diabetes care. 2010;33(2):414-20.
16. Cappuccio FP, D'Elia L, Strazzullo P, Miller MA. Sleep duration and all-cause mortality: a systematic review and meta-analysis of prospective studies. Sleep. 2010;33(5):585-92.
17. Hoevenaar-Blom MP, Spijkerman AM, Kromhout D, van den Berg JF, Verschuren W. Sleep duration and sleep quality in relation to 12-year

For peer review only - http://bmjopen.bmj.com/site/about/guidelines.xhtml

cardiovascular disease incidence: the MORGEN study. Sleep. 2011;34(11):1487-92.
18. Lou P, Chen P, Zhang L, Zhang P, Yu J, Zhang N, et al. Relation of sleep quality and sleep duration to type 2 diabetes: a population-based cross-sectional survey. BMJ open. 2012;2(4):e000956.
19. Ford DE, Kamerow DB. Epidemiologic study of sleep disturbances and psychiatric disorders: an opportunity for prevention? Jama. 1989;262(11):1479-84.
20. Patel SR, Blackwell T, Redline S, Ancoli-Israel S, Cauley JA, Hillier TA, et al. The association between sleep duration and obesity in older adults. International journal of obesity. 2008;32(12):1825.
21. Casarett D, Karlawish J, Sankar P, Hirschman K, Asch DA. Designing pain research from the patient's perspective: what trial end points are important to patients with chronic pain? Pain Medicine. 2001;2(4):309-16.
22. Turk DC, Dworkin RH, Revicki D, Harding G, Burke LB, Cella D, et al. Identifying important outcome domains for chronic pain clinical trials: an IMMPACT survey of people with pain. PAIN®. 2008;137(2):276-85.
23. Krystal AD. A compendium of placebo-controlled trials of the risks/benefits of pharmacological treatments for insomnia: the empirical basis for US clinical practice. Sleep medicine reviews. 2009;13(4):265-74.
24. de Gage SB, Bégaud B, Bazin F, Verdoux H, Dartigues J-F, Pérès K, et al. Benzodiazepine use and risk of dementia: prospective population based study. Bmj. 2012;345:e6231.
25. Glass J, Lanctôt KL, Herrmann N, Sproule BA, Busto UE. Sedative hypnotics in older people with insomnia: meta-analysis of risks and benefits. Bmj. 2005;331(7526):1169.
26. Kripke DF, Langer RD, Kline LE. Hypnotics' association with mortality or cancer: a matched cohort study. BMJ open. 2012;2(1):e000850.
27. Modén B, Merlo J, Ohlsson H, Rosvall M. Psychotropic drugs and falling accidents among the elderly: a nested case control study in the whole population of Scania, Sweden. Journal of Epidemiology & Community Health. 2010;64(5):440-6.
28. Stewart R, Besset A, Bebbington P, Brugha T, Lindesay J, Jenkins R, et al. Insomnia comorbidity and impact and hypnotic use by age group in a national survey population aged 16 to 74 years. Sleep. 2006;29(11):1391-7.
29. Jann M, Kennedy WK, Lopez G. Benzodiazepines: a major component in unintentional prescription drug overdoses with opioid analgesics. Journal of pharmacy practice. 2014;27(1):5-16.
30. Montgomery P, Dennis J. A systematic review of non-pharmacological therapies for sleep problems in later life. Sleep medicine reviews. 2004;8(1):47-62.
31. Morgenthaler T, Kramer M, Alessi C, Friedman L, Boehlecke B, Brown T, et al. Practice parameters for the psychological and behavioral treatment of insomnia: an update. An american academy of sleep medicine report. Sleep. 2006;29(11):1415.
32. MORIN C, CULBERT J, SCHWARTZ S. Nonpharmacological interventions for insomnia: a meta-analysis of treatment efficacy. The American journal of psychiatry. 1994;151(8):1172-80.

For peer review only

33. Sateia M, Doghramji K, Hauri P, Morin C. Evaluation of chronic insomnia. An American Academy of Sleep Medicine review. Sleep. 2000;23(2):243-308.
34. Trauer JM, Qian MY, Doyle JS, Rajaratnam SM, Cunnington D. Cognitive behavioral therapy for chronic insomnia: a systematic review and meta-analysis. Annals of internal medicine. 2015;163(3):191-204.
35. Tang NK, Lereya ST, Boulton H, Miller MA, Wolke D, Cappuccio FP. Nonpharmacological treatments of insomnia for long-term painful conditions: a systematic review and meta-analysis of patient-reported outcomes in randomized controlled trials. Sleep. 2015;38(11):1751-64.
36. Wilson S, Anderson K, Baldwin D, Dijk D-J, Espie A, Espie C, et al. British Association for Psychopharmacology consensus statement on evidence-based treatment of insomnia, parasomnias and circadian rhythm disorders: An update. Journal of Psychopharmacology. 2019:0269881119855343.
37. Tang NK. Cognitive-behavioral therapy for sleep abnormalities of chronic pain patients. Current rheumatology reports. 2009;11(6):451.
38. Excellence NIfC. Guidance on the use of zaleplon, zolpidem and zopiclone for the short-term management of insomnia. 2004. Technology Appraisal Guidance.77.
39. Mack LJ, Rybarczyk BD. Behavioral treatment of insomnia: a proposal for a stepped-care approach to promote public health. Nature and science of sleep. 2011;3:87.
40. Perlis ML, Smith MT. How can we make CBT-I and other BSM services widely available? Journal of Clinical Sleep Medicine. 2008;4(01):11-3.
41. Gordon K, Rice H, Allcock N, Bell P, Dunbar M, Gilbert S, et al. Barriers to self-management of chronic pain in primary care: a qualitative focus group study. Br J Gen Pract. 2017;67(656):e209-e17.
42. Tang NK, Goodchild CE, Salkovskis PM. Hybrid cognitive-behaviour therapy for individuals with insomnia and chronic pain: a pilot randomised controlled trial. Behaviour research and therapy. 2012;50(12):814-21.
43. Berger AM, Kuhn BR, Farr LA, Von Essen SG, Chamberlain J, Lynch JC, et al. One-year outcomes of a behavioral therapy intervention trial on sleep quality and cancer-related fatigue. Journal of Clinical Oncology. 2009;27(35):6033.
44. Öst L-G, Ollendick TH. Brief, intensive and concentrated cognitive behavioral treatments for anxiety disorders in children: A systematic review and meta-analysis. Behaviour research and therapy. 2017;97:134-45.
45. Bevan A, Oldfield VB, Salkovskis PM. A qualitative study of the acceptability of an intensive format for the delivery of cognitive - behavioural therapy for obsessive - compulsive disorder. British Journal of Clinical Psychology. 2010;49(2):173-91.
46. Morgan K, Gregory P, Tomeny M, David BM, Gascoigne C. Self - help treatment for insomnia symptoms associated with chronic conditions in older adults: A randomized controlled trial. Journal of the American Geriatrics Society. 2012;60(10):1803-10.
47. Espie CA, Inglis SJ, Tessier S, Harvey L. The clinical effectiveness of cognitive behaviour therapy for chronic insomnia: implementation and evaluation of a sleep clinic in general medical practice. Behaviour research and therapy. 2001;39(1):45-60.
48. Public Health England. National General Practice Profiles 2019 [Available from: https://fingertips.phe.org.uk/profile/general-practice.

For peer review only - http://bmjopen.bmj.com/site/about/guidelines.xhtml

49. Edinger JD, Bonnet MH, Bootzin RR, Doghramji K, Dorsey CM, Espie CA, et al. Derivation of Research Diagnostic Criteria for Insomnia: Report of an American Academy of Sleep Medicine Work Group. Sleep. 2004;27(8):1567-96.
50. American Psychiatric Association. Diagnostic and statistical manual of mental disorders 5th ed. Washington, D.C.: American Psychiatric Association; 2013.
51. Bastien CH, Vallières A, Morin CM. Validation of the Insomnia Severity Index as an outcome measure for insomnia research. Sleep medicine. 2001;2(4):297-307.
52. Cleeland CS, Chapman CR, Loeser JD. Measurement of pain by subjective report. In: Chapman CR, Loeser JD, editors. Advances in Pain Research and Therapy, Volume 12: Issues in Pain Measurement. New York: Raven Press; 1989. p. 391 - 403.
53. Smets E, Garssen B, Bonke Bd, De Haes J. The Multidimensional Fatigue Inventory (MFI) psychometric qualities of an instrument to assess fatigue. Journal of psychosomatic research. 1995;39(3):315-25.
54. Zigmond AS, Snaith RP. The hospital anxiety and depression scale. Acta psychiatrica scandinavica. 1983;67(6):361-70.
55. Group TE. EuroQol-a new facility for the measurement of health-related quality of life. Health policy. 1990;16(3):199-208.
56. Devlin NJ, Shah KK, Feng Y, Mulhern B, van Hout B. Valuing health ‐ related quality of life: An EQ ‐ 5 D ‐ 5 L value set for E ngland. Health economics. 2018;27(1):7-22.
57. Sullivan MJ, Bishop SR, Pivik J. The pain catastrophizing scale: development and validation. Psychological assessment. 1995;7(4):524.
58. Tang NK, Salkovskis PM, Hanna M. Mental defeat in chronic pain: initial exploration of the concept. The Clinical journal of pain. 2007;23(3):222-32.
59. Tang NK, Harvey AG. Correcting distorted perception of sleep in insomnia: a novel behavioural experiment? Behaviour research and therapy. 2004;42(1):27-39.
60. Morin CM, Vallières A, Ivers H. Dysfunctional beliefs and attitudes about sleep (DBAS): validation of a brief version (DBAS-16). Sleep. 2007;30(11):1547-54.
61. Afolalu EF, Moore C, Ramlee F, Goodchild CE, Tang NK. Development of the pain-related beliefs and attitudes about sleep (PBAS) scale for the assessment and treatment of insomnia comorbid with chronic pain. Journal of Clinical Sleep Medicine. 2016;12(09):1269-77.
62. Carney CE, Buysse DJ, Ancoli-Israel S, Edinger JD, Krystal AD, Lichstein KL, et al. The consensus sleep diary: standardizing prospective sleep self-monitoring. Sleep. 2012;35(2):287.
63. Braun V, Clarke V. Using thematic analysis in psychology. Qualitative research in psychology. 2006;3(2):77-101.
64. Johannes CB, Le TK, Zhou X, Johnston JA, Dworkin RH. The Prevalence of Chronic Pain in United States Adults: Results of an Internet-Based Survey. The Journal of Pain. 2010;11(11):1230-9.
65. Morin CM, Benca R. Chronic insomnia. The Lancet. 2012;379(9821):1129-41.

For peer review only

66. Morin CM, LeBlanc M, Daley M, Gregoire JP, Mérette C. Epidemiology of insomnia: Prevalence, self-help treatments, consultations, and determinants of help-seeking behaviors. Sleep Medicine. 2006;7(2):123-30.
67. van Hecke O, Torrance N, Smith BH. Chronic pain epidemiology and its clinical relevance. British Journal of Anaesthesia. 2013;111(1):13-8.
68. Stinson K, Tang NKY, Harvey AG. Barriers to treatment seeking in primary insomnia in the United Kingdom: a cross-sectional perspective. Sleep. 2006;29(12):1643-6.
69. Vitiello MV, McCurry SM, Shortreed SM, Balderson BH, Baker LD, Keefe FJ, et al. Cognitive - behavioral treatment for comorbid insomnia and osteoarthritis pain in primary care: The lifestyles randomized controlled trial. Journal of the American Geriatrics Society. 2013;61(6):947-56.
70. ISRCTN42499563. Health professional Administered Brief Insomnia Therapy (HABIT) trial 2019 [Available from: http://www.isrctn.com/ISRCTN42499563?q=&filters=&sort=&offset=2&totalResults=17076&page=1&pageSize=10&searchType=basic-search.
71. Van Straten A, Cuijpers P. Self-help therapy for insomnia: a meta-analysis. Sleep medicine reviews. 2009;13(1):61-71.

For peer review only

Box 1. Seed questions used in the semi-structured interviews pre- and post-intervention for both Hybrid CBT group and Self-help control group arms.

**A. Pre-intervention interview**
1. We are very interested in your journey. When did you first notice your pain-related sleep disturbance?
2. Did you put it down to anything?
3. Did you talk to anyone about it?
4. What did you do to try to help?
5. Does anything make it better or worse?
6. Have you seen any health professionals or other practitioners?
7. What do you think is going to happen in this study?
8. What are you hoping to get from this study?

**B. Post-intervention interview**
1. How did you get on with the study?
2. Did you get any benefit from the study (attending the sessions/receiving the booklets)?
3. What worked for you?
4. What did not work for you?
5. Would you recommend this type of programme to other people?
6. If we ran this programme in a larger study is there anything you would change?
7. How did you get on with the paperwork?
8. How did you find the process of being in a research study?

For peer review only

Table 1. Participants characteristics as measured at baseline.

**Baseline variable**		**All N=25**
Recruitment centre (n, %)	Primary Care Centre 1	8 (32)
	Primary Care Centre 2	10 (40)
	Primary Care Centre 3	7 (28)
Gender (n, %)	Female	14 (56.0)
Age: years (mean, SD)		49.3 (9.8) †
BMI: kg/m^2 (mean, SD)		29.3 (7.6)
Ethnicity (n, %)	White	24 (96)
Relationship status (n, %)	Cohabiting/ Married/Engaged	15 (60)
	Single/ Separating	10 (40)
Education (n, %)	No formal qualifications	8 (32)
	Secondary	11 (44)
	Degree/professional qualification	6 (24)
Employment (n, %)	Paid work	10 (40)
	Retired/medically retired	6 (24)
	Unemployed	5 (20)
	Other	4 (16)
Receiving benefits? (n, %)	Yes	9 (36) †
How long have you had the pain (minimum, in years) (mean, sd)		11.0 (9.0) ‡
What the pain is like (n, %)	Constant	20 (80)
	Recurrent	3 (12)
	Occasional	1 (4)
	Missing	1 (4)
No. of painful places given (median, range)		5 (1 – 8)
Where is the pain (n, %)	Head	3 (12)
	Neck	17 (68)
	Shoulders	18 (68)
	Upper Back	6 (24)
	Lower back	18 (72)
	Arms	9 (36)
	Legs	15 (60)
	Knees	13 (52)
	Abdomen	2 (8)
	Joints	15 (60)
	Other	7 (28)
Brief Pain Inventory (mean, sd)	Current pain severity	6.1 (1.5)
	Current pain interference	6.6 (1.5)
Insomnia Severity Index (mean, sd)	Total score	20.1 (4.9)

† 1 participant missing data. ‡ 3 participants missing data.

For peer review only

Table 2. Descriptive statistics of the candidate primary and secondary outcome measures, as well as process measures.

	Baseline		12 weeks		24 weeks	
	All (n=25)	No. of valid response (n, %)	All (n=25)	No. of valid response (n, %)	All (n=25)	No. of valid response (n, %)
Primary outcomes						
ISI (mean, sd)	20.2 (4.7)	24 (96)	14.4 (10.3)	10 (40)	14.8 (11.8)	8 (32)
BPI interference	6.1 (1.7)	24 (96)	4.9 (2.5)	12 (48)	4.7 (2.7)	8 (32)
Secondary outcomes						
BPI - pain intensity	6.2 (1.6)	24 (96)	5.1 (2.4)	12 (48)	5.5 (2.6)	8 (32)
MFI - general fatigue	16.3 (3.6)	24 (96)	13.8 (3.7)	12 (48)	14.9 (3.2)	8 (32)
HAD - anxiety	9.7 (3.0)	24 (96)	6.7 (5.5)	12 (48)	8.9 (4.9)	8 (32)
HAD - depression	8.4 (3.5)	24 (96)	7.3 (4.6)	12 (48)	7.8 (5.1)	8 (32)
EQ-5D - health thermometer score	50 (17.5)	24 (96)	58.9 (15.2)	12 (48)	58.8 (23.0)	8 (32)
EQ-5D - utility score	0.60 (0.19)	24 (96)	0.57 (0.29)	12 (48)	0.56 (0.33)	8 (32)
Process measures						
PCS - pain catastrophising	15.7 (9.2)	24 (96)	9.5 (8.5)	12 (48)	10 (8.6)	8 (32)
PSPS - mental defeat	30.8 (23.6)	23 (92)	21.9 (27.3)	12 (48)	14.7 (21)	6 (24)
APSQ - sleep anxiety	68 (21.7)	24 (96)	50.2 (32.4)	12 (48)	49.2 (37)	9 (36)
DBAS - sleep beliefs	5.6 (2.1)	21 (84)	4.1 (2.9)	10 (40)	4.0 (3.3)	8 (32)
PBAS –-pain-related sleep beliefs	7.1 (2.1)	21 (84)	5.1 (3.4)	12 (48)	5.0 (3.7)	9 (36)

Notes. ISI = Insomnia Severity Index. BPI = Brief Pain Inventory. MFI = Multidimensional Fatigue Inventory. HAD = Hospital Anxiety and Depression Scale. EQ-5D = EuroQol EQ-5D-5L. PCS = Pain Catastrophizing Scale. PSPS = Pain Self Perception Scale. APSQ = Anxiety and Preoccupation about Sleep Questionnaire. DBAS = Dysfunctional Beliefs and Attitudes and Sleep scale. PBAS = Pain-related Dysfunctional Beliefs and Attitudes about Sleep scale.

For peer review only

Table 3. A summary of findings from the pre-intervention interviews.

Theme	Key points about the theme	Example quotes and/or additional notes* (Patient code for example quotes e.g., *Patient X*)	Implications for treatment refinement and future trial planning
Pain changed who I am 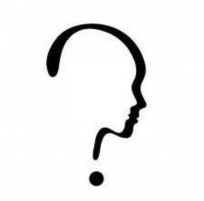	Participants define themselves by their experience of pain and losses due to pain.<br><br>Frequent comparisons were made of what life was in the past, with what life is now and what life should be like at a certain age.<br><br>The way in which participants spoke of their struggle with pain carried a sense of mental defeat.	*"So generally you're in pain the whole time…and [you] can't, literally can't move and somebody's got to help you out of bed, which really at 48 I'm not being funny, I shouldn't be like that. … I feel I'm always going to be a person with pain, there's no debate on that as far as I'm aware…I don't see any way of resolving these issues."* (Patient D)<br><br>*"I've been suffering with sleep deprivation for many years, um… pain the same. … So yeah it destroyed everything really, destroyed my career, destroyed… when [you're] miserable with pain and stuff nobody wants you to be in a relationship with [you], so the relationships break up, and it becomes too much for them, and I understand um so yeah life changed a hell of a lot, it did, it changed um… it's like it stole my life away."* (Patient C)<br><br>*"I like to think that I'm quite strong but equally I feel that…I've given up. I feel like I'm*	Patients' damaged sense of identity, and the related psychological processes that feed into it, should be a core target of treatment.

For peer review only

		giving up again… I'm frustrated 'cause I'm hurting. I can't escape. I'm getting it, brain is just struggling [to] absorb and cope with things, and then you worry about your future and how am I gonna. How things gonna turn out… Is it gonna keep falling down down down down?" (Patient E)	
Pain and sleep did not occur in psychosocial vacuum 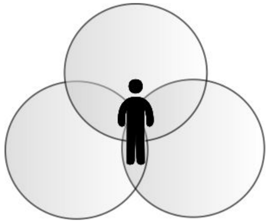	Participants' descriptions of their treatment journeys made it clear that the issues of chronic pain and insomnia were embedded within a larger context of personal adversities.<br><br>Specifics of these adversities* revealed themselves at different places of the interviews, where details of personal lives were volunteered to situate the conversation. Adversities came not in isolation but in clusters as chronic pain and insomnia became increasingly more severe and disabling.<br><br>Challenging to accurately tell whether these were psychosocial triggers or consequences of chronic pain and insomnia.	* *No one single quote could satisfactorily illustrate the complexity of the psychosocial contexts described by the participants, and without risks of revealing their identities. Example adversities cited included ill health, mental health problems, car or work accidents, assaults, relationship breakdown, problems experienced by dependents or close family members, being a carer, job redundancy/ unemployment, financial difficulties, homelessness, and bereavement.*	More consideration should be given to the context in which the treatment is being delivered, as well as to practical support required to enable the most disadvantaged/burdened patients to access treatment.

For peer review only

Theme	Key points about the theme	Example quotes and/or additional notes* (Patient code for example quotes e.g., *Patient X*)	Implications for treatment refinement and future trial planning
Participants were not treatment naïve<br>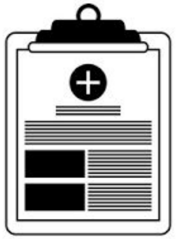	Perhaps an artefact of self-selection bias in an RCT; participants were desperate to engage in self-help to get better, so they could get on with their lives.<br><br>All had tried to manage their pain and insomnia using a combination of drug and non-drug strategies, invariably with limited success.<br><br>The self-helping spirit is a double-edge sword. If the right treatment is identified, it could facilitate engagement and maximise treatment gains. But it could also drive people to take bold steps to keep pain and sleep problems under control, using strategies that are not necessarily recommended by current evidence-based guidelines, which may lead to dashed hope and further demoralisation.	*"You just try to help yourself a little bit but, whether that's a good or bad thing I don't know."* (Patient A)<br><br>*"…Now I try to keep [pain medication] in my system all the time so there's always something there…to take the edge off…rather than waiting for the pain to start and then taking [the medication]. [Interviewer: Right so have you done that on the advice of somebody or off your own back?] No just off my own back, just to try and keep it under control a little bit."* (Patient B)<br><br>*"I'm trying to think myself healthy…I've tried you know the thing with sleep, you know I've tried like you know books and this and having your room right and spraying your pillow and you know and all sorts of things…"* (Patient D)<br><br>*"…I'm a person who always absorbs information. That's always been the quest of my life anyway, so, there wasn't nothing that I haven't already seen or read or something*	Self-help treatments may not be considered as a satisfactory treatment option by this non-treatment naïve clinical population.<br><br>A wait-list control or an active alternative treatment may be a more appropriate control intervention.

For peer review only - http://bmjopen.bmj.com/site/about/guidelines.xhtml

		before, it wasn't the fact that your stuff's wrong, it's just I'm good at doing my side of things…But that's [because] they haven't done what I've already done, is try to self-help already." (Patient E)	
Pain was thought to be the primary cause of sleep problems 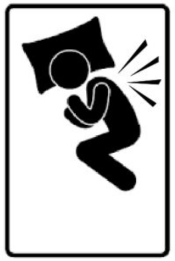	All participants shared a strong belief that pain was a major cause of their sleep problems. However, there was the awareness that other factors might also play a role in aggravating the sleep problems and that not sleeping well could have a reciprocal effect on the pain.	*"Then yeah that's sleep it's gone out of the window 'cause, as soon as I get uncomfortable or in pain I'm awake…Popping pain killers like no tomorrow then... [It's the pain that] keeps me awake…As soon as you have pain then wakes…Sometimes you can't sleep because of the pain, and then you're up all night, you get yourself angry with yourself…"* (Patient C)<br><br>*"I'm not just gonna blame the pain…I've got a sixteen year old son who's causing you know he's a lot of issues, and I really don't think you know, I'm not naïve to think that's not a contributing factor [to sleep problems]… And I do stress you know, I think that's just in my nature."* (Patient D)<br><br>*"So really personal circumstances have made a major effect… I found that 'cause I wasn't sleeping the pain seemed more unbearable… Unbearable, just 'cause I was tired… And I felt run down it just seemed worse I think…"* (Patient E)	If patients hold a rigid belief that sleep will never improve unless pain is resolved, it would be difficult to get their buy-in to the Hybrid CBT on offer. As such, these beliefs need to be addressed upfront to improve treatment uptake and subsequent adherence.

For peer review only

Theme	Key points about the theme	Example quotes and/or additional notes* (Patient code for example quotes e.g., *Patient X*)	Implications for treatment refinement and future trial planning
Participants were dissatisfied with the services available 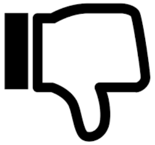	All participants interviewed have had much interaction with multiple health service providers.<br><br>They were most frustrated when they felt they were not being listened to or misunderstood by their GPs.<br><br>They were also not happy with a lack of effective treatment choices, noting a contradictory combination of a heavy reliance on drugs for pain control with a general reluctance to prescribe sleeping tablets for insomnia.	*"I kept going with all this pain problems and that, never get nowhere… Just felt as though it was all in my head, nobody would listen to me, hand me one and you know, here she goes again (laughs) people do, 'cause they can't see nothing you see?"* (Patient A)<br><br>*"Most doctors these days don't…They don't give the time of day. They've got your prescription written out before you go in."* (Patient C)<br><br>*"[Interviewer: So have you talked to anybody about your pain and sleep?] Only my GP…And they sort of got painkillers. They don't really like to give sleeping tablets anymore. Um... They advised over the counter ones...which work to an extent but I've found if you take one one night, take it again the next night it doesn't work…So you sort of have to leave a few nights in between so you can't take them every night."* (Patient B)<br><br>*"I don't feel that this surgery offers a lot of that [non-drug treatments]. Yeah it can give*	The issue of validation (or the lack thereof) is not unique to chronic pain patients, but highlights the importance for future trials to provide generic clinical skills training to the study therapists (health psychologists in the current study, or other suitably trained allied health care professionals with appropriate expertise in future trials).

For peer review only

		ibuprofen, it can give me (a medicine name) or it can in the past give me, um tramadol, but obviously I've stopped taking all tablets now for five weeks and I can't see if there's a difference from taking tablets to the placebo effect of fear that at least by reaching for the tablet there was something to help me." (Patient E)	
Participants' treatment expectations were high 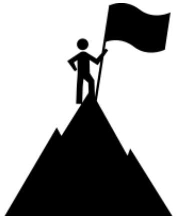	Participants interviewed showed understanding that the interventions offered were not 'magic cures', but nonetheless had high expectations for the treatments, reflecting the constant tension between ideally what the patients want ("no pain" and "lots of sleep") and realistically what can be offered by an intervention designed to optimise management of these problems.	*"Well just to help control pain and sleeping…you can't work miracles but it might be something that can help me…To be honest I don't, I'm hoping with (laughs) something, you might have the magic cure, you never know."* (Patient A)<br><br>*"A bit more sleep. More than anything. I find if I'm tired, I don't know if the pain seems worse or I'm just not able to cope with it as well…So my theory is if I can just get a bit more sleep I can perhaps cope better with the pain."* (Patient B)<br><br>*"Obviously it's not going to be magical overnight but even if it could give me ideas if I'm in that position how I should be um… reacting, you know um… I'm willing to try…. Well my future…hopefully I will have no pain, no sleep, lots of sleep not no sleep (laughs)… Lots of sleep and no stress."* (Patient D)	Proactive management of patients' treatment expectations at the outset of treatment may help minimise attrition and unnecessary demoralisation.

For peer review only

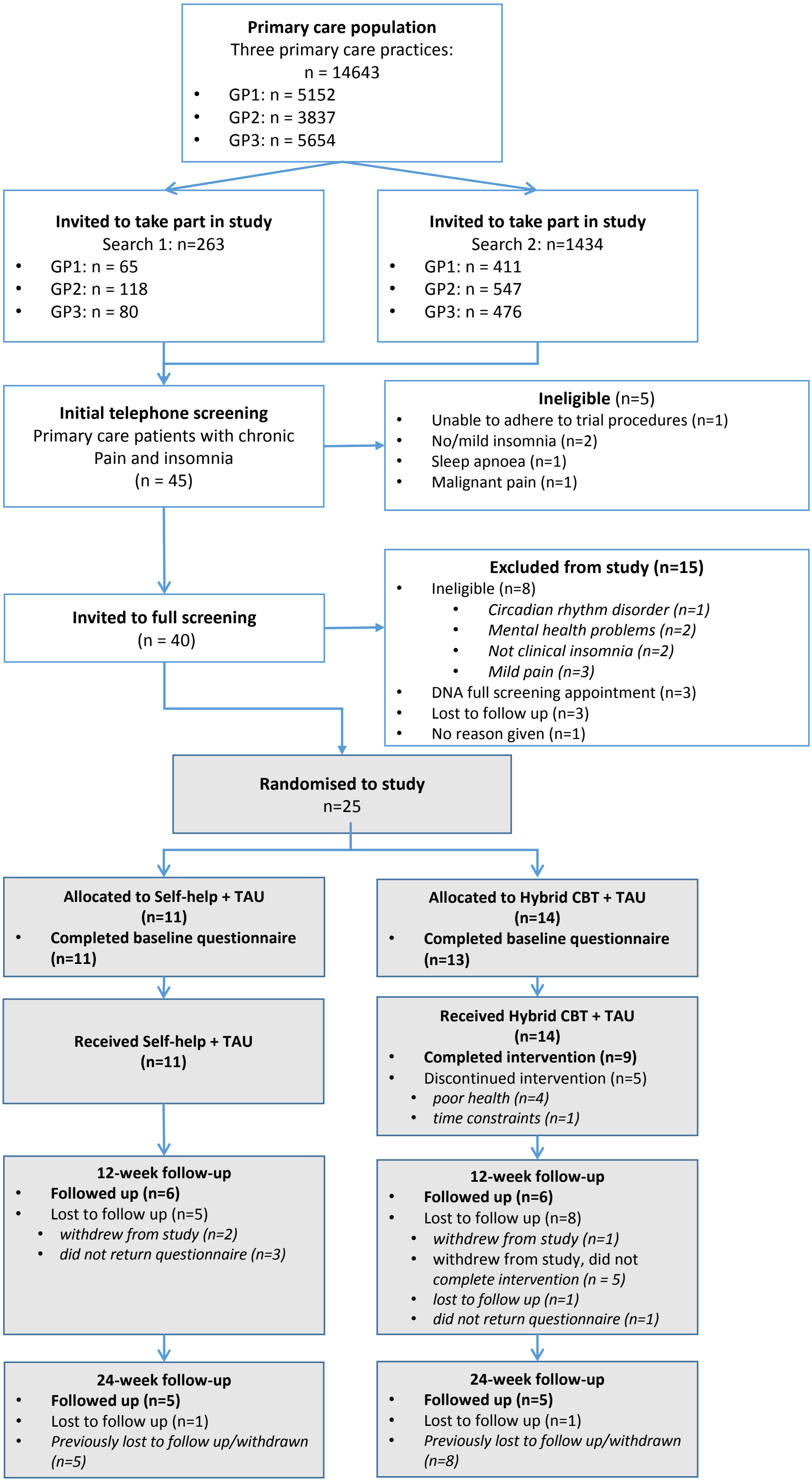

Figure 1. CONSORT flow diagram from screening (open boxes) to post-screening (filled boxes) processes in the study.

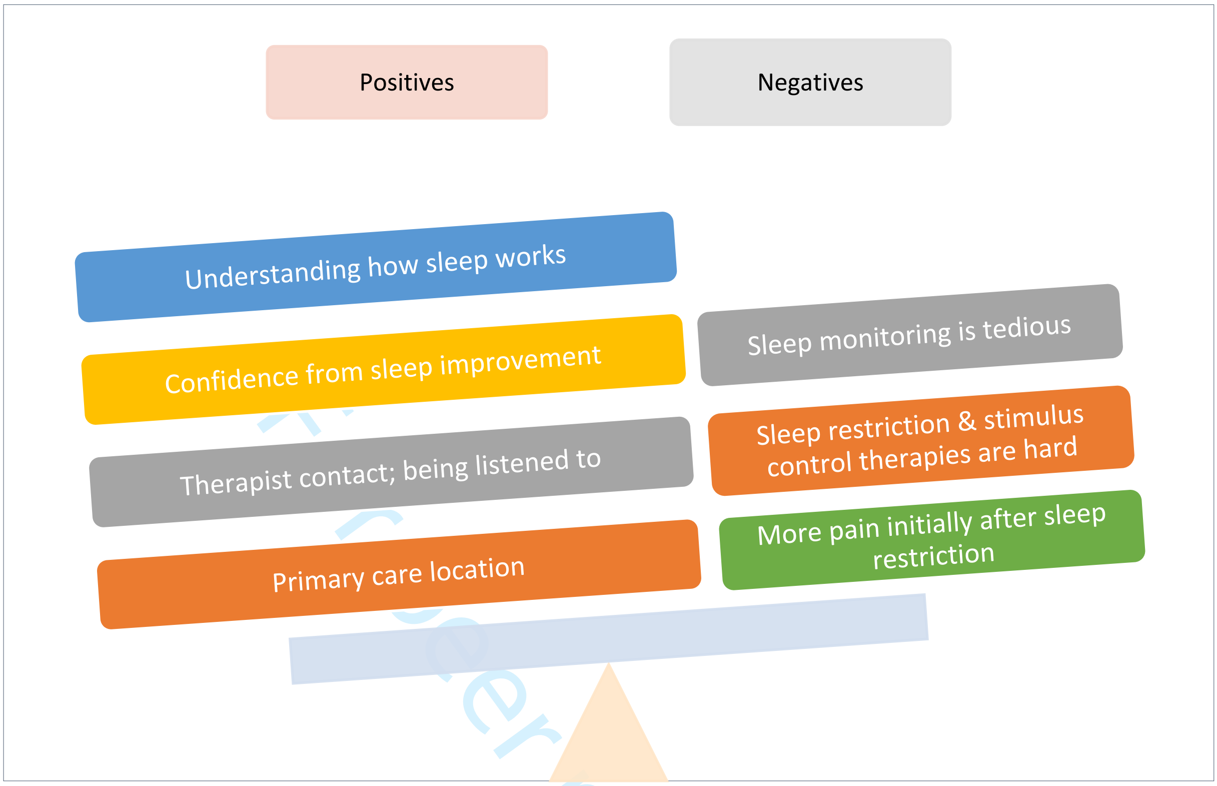

Figure 2. A summary of themes from post-intervention interviews of Hybrid CBT participants, highlighting the positives and negatives of the current treatment approach and content.

CONSORT 2010 checklist of information to include when reporting a randomised trial*

Section/Topic	Item No	Checklist item	Reported on page No
**Title and abstract**			
	1a	Identification as a randomised trial in the title	N/A (mixed-methods feasibility study)
	1b	Structured summary of trial design, methods, results, and conclusions (for specific guidance see CONSORT for abstracts)	3
**Introduction**			
Background and objectives	2a	Scientific background and explanation of rationale	6-7
	2b	Specific objectives or hypotheses	7
**Methods**			
Trial design	3a	Description of trial design (such as parallel, factorial) including allocation ratio	8
	3b	Important changes to methods after trial commencement (such as eligibility criteria), with reasons	N/A
Participants	4a	Eligibility criteria for participants	10-11
	4b	Settings and locations where the data were collected	8, 10
Interventions	5	The interventions for each group with sufficient details to allow replication, including how and when they were actually administered	8-9
Outcomes	6a	Completely defined pre-specified primary and secondary outcome measures, including how and when they were assessed	11-12, 12-13
	6b	Any changes to trial outcomes after the trial commenced, with reasons	N/A
Sample size	7a	How sample size was determined	11
	7b	When applicable, explanation of any interim analyses and stopping guidelines	N/A
Randomisation:			
Sequence generation	8a	Method used to generate the random allocation sequence	11
	8b	Type of randomisation; details of any restriction (such as blocking and block size)	11
Allocation concealment mechanism	9	Mechanism used to implement the random allocation sequence (such as sequentially numbered containers), describing any steps taken to conceal the sequence until interventions were assigned	11

For peer review only - http://bmjopen.bmj.com/site/about/guidelines.xhtml

Implementation	10	Who generated the random allocation sequence, who enrolled participants, and who assigned participants to interventions	11
Blinding	11a	If done, who was blinded after assignment to interventions (for example, participants, care providers, those assessing outcomes) and how	11
	11b	If relevant, description of the similarity of interventions	8-9
Statistical methods	12a	Statistical methods used to compare groups for primary and secondary outcomes	11, 12
	12b	Methods for additional analyses, such as subgroup analyses and adjusted analyses	12-13
**Results**			
Participant flow (a diagram is strongly recommended)	13a	For each group, the numbers of participants who were randomly assigned, received intended treatment, and were analysed for the primary outcome	14 and Fig.1
	13b	For each group, losses and exclusions after randomisation, together with reasons	13-14, Fig.1
Recruitment	14a	Dates defining the periods of recruitment and follow-up	10
	14b	Why the trial ended or was stopped	10
Baseline data	15	A table showing baseline demographic and clinical characteristics for each group	Table 1
Numbers analysed	16	For each group, number of participants (denominator) included in each analysis and whether the analysis was by original assigned groups	Tables 1 & 2 (reported all participants due to small n and the feasibility nature of the study) and in text for the qualitative analysis pp. 15-19
Outcomes and estimation	17a	For each primary and secondary outcome, results for each group, and the estimated effect size and its precision (such as 95% confidence interval)	Table 2; focus on study not on effect size
	17b	For binary outcomes, presentation of both absolute and relative effect sizes is recommended	N/A
Ancillary analyses	18	Results of any other analyses performed, including subgroup analyses and adjusted analyses, distinguishing pre-specified from exploratory	N/A
Harms	19	All important harms or unintended effects in each group (for specific guidance see CONSORT for harms)	15, 22

For peer review only - http://bmjopen.bmj.com/site/about/guidelines.xhtml

For peer review only

**Discussion**			
Limitations	20	Trial limitations, addressing sources of potential bias, imprecision, and, if relevant, multiplicity of analyses	21-22
Generalisability	21	Generalisability (external validity, applicability) of the trial findings	19-22
Interpretation	22	Interpretation consistent with results, balancing benefits and harms, and considering other relevant evidence	19-22
**Other information**			
Registration	23	Registration number and name of trial registry	Abstract & p8
Protocol	24	Where the full trial protocol can be accessed, if available	Abstract & p8
Funding	25	Sources of funding and other support (such as supply of drugs), role of funders	Cover pages

*We strongly recommend reading this statement in conjunction with the CONSORT 2010 Explanation and Elaboration for important clarifications on all the items. If relevant, we also recommend reading CONSORT extensions for cluster randomised trials, non-inferiority and equivalence trials, non-pharmacological treatments, herbal interventions, and pragmatic trials. Additional extensions are forthcoming: for those and for up to date references relevant to this checklist, see www.consort-statement.org.

For peer review only

For peer review only - http://bmjopen.bmj.com/site/about/guidelines.xhtml

Consolidated criteria for reporting qualitative studies (COREQ): 32-item checklist

No	Item	Guide questions/description	Reported on Page No.
**Domain 1: Research team and reflexivity**			
	Personal Characteristics		
1.	Interviewer/facilitator	Which author/s conducted the interview or focus group?	12
2.	Credentials	What were the researcher's credentials? *E.g. PhD, MD*	1, 12
3.	Occupation	What was their occupation at the time of the study?	12
4.	Gender	Was the researcher male or female?	N/A (gender neutral language was used)
5.	Experience and training	What experience or training did the researcher have?	12
	Relationship with participants		
6.	Relationship established	Was a relationship established prior to study commencement?	12
7.	Participant knowledge of interviewer	What did the participants know about the researcher? *E.g. personal goals, reasons for doing the research.*	12
8.	Interviewer characteristics	What characteristics were reported about the interviewer/facilitator? *E.g. Bias, assumptions, reasons and interests in the research topic.*	12
**Domain 2: Study design**			
	Theoretical Framework		
9.	Methodological orientation and theory	What methodological orientation was stated to underpin the study? *E.g. grounded theory, discourse analysis, ethnography, phenomenology, content analysis*	12
	Participant Selection		
10.	Sampling	How were participants selected? *E.g. purposive, convenience, consecutive, snowball*	12
11.	Method of approach	How were participants approached? *E.g. face-to-face, telephone, mail, email*	12
12.	Sample size	How many participants were in the study?	13-14, 15
13.	Non-participation	How many people refused to participate or dropped out? Reason?	13-14, 15

For peer review only - http://bmjopen.bmj.com/site/about/guidelines.xhtml

Consolidated criteria for reporting qualitative studies (COREQ): 32-item checklist

	Setting		
14.	Setting of data collection	Where was the data collected? *E.g. home, clinic, workplace*	12
15.	Presence of non-participants	Was anyone else present besides the participants and researchers?	N/A
16.	Description of sample	What are the important characteristics of the sample? *E.g. demographic data and date*	14 , 15 + Table 1, Table 3
	Data Collection		
17.	Interview guide	Were questions, prompts, guides provided by authors? Was it pilot tested?	14 + Box 1
18.	Repeat interviews	Were repeat interviews carried out? If yes, how many?	N/A; pre-post interview with different questions (p.14 + Box 1)
19.	Audio/visual recording	Did the research use audio or visual recording to collect the data?	13
20.	Field notes	Were field notes made during and/or after the interview or focus group?	13
21.	Duration	What was the duration of the interviews or focus group?	13
22.	Data saturation	Was data saturation discussed?	N/A
23.	Transcripts returned	Were transcripts returned to participants for comment and/or correction?	N/A
**Domain 3: Analysis and findings**			
	Data Analysis		
24.	Number of data coders	How many data coders coded the data?	13
25.	Description of the coding tree	Did authors provide a description of the coding tree?	N/A
26.	Derivation of themes	Were themes identified in advance or derived from the data?	13
27.	Software	What software, if applicable, was used to manage the data?	13
28.	Participant checking	Did participants provide feedback on the findings?	N/A
	Reporting		
29.	Quotations presented	Were participant quotations presented to illustrate the themes/findings? Was each quotation identified? *E.g. partipcipant number*	15-19; Table 3, Figure 2

For peer review only

Consolidated criteria for reporting qualitative studies (COREQ): 32-item checklist

30.	Data and findings consistent	Was there consistency between the data presented and the findings?	N/A
31.	Clarity of major themes	Were the major themes clearly presented in the findings?	Table 3 + Fig 2
32.	Clarity of minor themes	Is there a description of diverse cases or discussion of minor themes?	15-19

For peer review only

For peer review only - http://bmjopen.bmj.com/site/about/guidelines.xhtml

BMJ Open

Implementing a hybrid cognitive-behavioural therapy for pain-related insomnia in primary care: Lessons learned from a mixed-methods feasibility study

Journal:	*BMJ Open*
Manuscript ID	bmjopen-2019-034764.R1
Article Type:	Original research
Date Submitted by the Author:	29-Jan-2020

Complete List of Authors:	Tang, Nicole; University of Warwick, Psychology<br>Moore, Corran; University of Leicester, Psychology<br>Parsons, Helen; University of Warwick, Division of Health Sciences<br>Sandhu, Harbinder; University of Warwick Warwick Medical School, Clinical Trials Unit<br>Patel, Shilpa; Warwick University, Division of Health Sciences<br>Ellard, David; University of Warwick, Clinical Trials Unit;<br>Nichols, Vivien; University of Warwick, Warwick Clinical Trials Unit<br>Madan, Jason; University of Warwick, Warwick Medical School<br>Collard, Victoria; University of Warwick, Psychology<br>Sharma, Uma<br>Underwood, Martin; Warwick University, Warwick Medical School

**Primary Subject Heading**:	Complementary medicine
Secondary Subject Heading:	General practice / Family practice
Keywords:	PAIN MANAGEMENT, SLEEP MEDICINE, Sleep medicine < ANAESTHETICS, MENTAL HEALTH, PRIMARY CARE, QUALITATIVE RESEARCH

SCHOLARONE™
Manuscripts

I, the Submitting Author has the right to grant and does grant on behalf of all authors of the Work (as defined in the below author licence), an exclusive licence and/or a non-exclusive licence for contributions from authors who are: i) UK Crown employees; ii) where BMJ has agreed a CC-BY licence shall apply, and/or iii) in accordance with the terms applicable for US Federal Government officers or employees acting as part of their official duties; on a worldwide, perpetual, irrevocable, royalty-free basis to BMJ Publishing Group Ltd ("BMJ") its licensees and where the relevant Journal is co-owned by BMJ to the co-owners of the Journal, to publish the Work in this journal and any other BMJ products and to exploit all rights, as set out in our licence.

The Submitting Author accepts and understands that any supply made under these terms is made by BMJ to the Submitting Author unless you are acting as an employee on behalf of your employer or a postgraduate student of an affiliated institution which is paying any applicable article publishing charge ("APC") for Open Access articles. Where the Submitting Author wishes to make the Work available on an Open Access basis (and intends to pay the relevant APC), the terms of reuse of such Open Access shall be governed by a Creative Commons licence – details of these licences and which Creative Commons licence will apply to this Work are set out in our licence referred to above.

Other than as permitted in any relevant BMJ Author's Self Archiving Policies, I confirm this Work has not been accepted for publication elsewhere, is not being considered for publication elsewhere and does not duplicate material already published. I confirm all authors consent to publication of this Work and authorise the granting of this licence.

For peer review only

BMJ OPEN

Implementing a hybrid cognitive-behavioural therapy for pain-related insomnia in primary care: Lessons learned from a mixed-methods feasibility study

N. K. Y. Tang[1], C. Moore[1,4], H. Parsons[2, 3], H. Sandhu[2], S. Patel[2], D. R. Ellard[2], V.P. Nichols[2], J. Madan[2], V. E. J. Collard[1], U. Sharma[3], and M. Underwood[2, 5]

[1]Department of Psychology, University of Warwick, Coventry, UK

[2]Clinical Trials Unit, Warwick Medical School, University of Warwick, Coventry, UK

[3]Patient and Public Involvement representative

[4]School of Psychology, University of Leicester, Leicester, UK

[5] University Hospitals of Coventry and Warwickshire

Corresponding Author:

Nicole K. Y. Tang

Department of Psychology, University of Warwick

Gibbet Hill Road, Coventry, CV4 7AL, UK

Phone: +44 (0)2476150556

Email: n.tang@warwick.ac.uk

Keywords: Chronic pain, Insomnia, Sleep, Cognitive Behaviour(al)Therapy (CBT), Psychological Treatment, Hybrid CBT, Patient and Public Involvement (PPI)

Word counts: 5570 (with direct quotes), 4978 (without direct quotes)

No. of box, table, and figure: 1, 3, and 2

For peer review only

Acknowledgments

The authors thank the trial participants and the participating primary care centres for their help in patient recruitment.

Author Contributions

All authors have contributed to the interpretation of the data and manuscript preparation. NKYT was principal investigator and led the project. NKYT, HP, HS, SP, DE, JM US and MU were involved in the design of the study. NKYT, HS, SP led the treatment development and therapist training, with support from US. CM co-ordinated the recruitment of patients and data collection. VPN, DE, and VEJC conducted the qualitative interviews whilst HP conducted the quantitative data analysis.

Funding

This paper presents independent research funded by the NIHR under its Research for Patient Benefit (RfPB) Programme (Grant Reference Number PB-PG-0213-30121). The views expressed are those of the author(s) and not necessarily those of the NHS, the NIHR or the Department of Health and Social Care.

Competing interests

All authors have completed the ICMJE uniform disclosure form. HS & SP are directors of Health Psychology Services Ltd which in part provides psychological treatments for those with chronic pain. HS is chief investigator and co-investigator on other NIHR funded projects. MU was Chair of the NICE accreditation advisory committee until March 2017 for which he received a fee. He is chief investigator or co-investigator on multiple previous and current research grants from the UK National Institute for Health Research, Arthritis Research UK and is a co-investigator on grants funded by the Australian NHMRC. He is an NIHR Senior Investigator. He has received travel expenses for speaking at conferences from the professional organisations hosting the conferences. He is a director and shareholder of Clinvivo Ltd that provides electronic data collection for health services research. He is part of an academic partnership with Serco Ltd related to return to work initiatives. He is a co-investigator on a study receiving support in

For peer review only - http://bmjopen.bmj.com/site/about/guidelines.xhtml

kind from Stryker Ltd. He has accepted honoraria for teaching/lecturing from CARTA & Sterling Events. He is an editor of the NIHR journal series, and a member of the NIHR Journal Editors Group, for which he receives a fee. NKYT received grant funding as co-investigator from NIHR for other projects and current funding from the Medical Research Council as principal investigator. NKYT is also a member of the trial steering committee of an ongoing trial funded by the NIHR.

Ethics approval

The National Health Service (NHS) ethical approval was given by the National Research Ethics Service (NRES) Committee West Midlands – Solihull, REC Number: 14/WM/1053. All participants gave informed consent before commencing the study.

Data sharing statement

All data relevant to the study are included in the article or uploaded as supplementary information.

Open Access

For peer review only

Abstract

Objectives: To test the feasibility of implementing a brief but intensive hybrid cognitive behaviour therapy (Hybrid CBT) for pain-related insomnia

Design: Mixed-methods, with qualitative process evaluation on a two-arm randomised controlled feasibility trial

Setting: Primary care

Participants: Twenty-five adult patients with chronic pain and insomnia

Intervention: Hybrid CBT or Self-help control intervention

Primary and secondary outcome measures: Primary outcomes measures were the Insomnia Severity Index and interference scale of the Brief Pain Inventory (BPI). Secondary outcomes measures were the present pain intensity rating from the BPI, Multidimensional Fatigue Inventory, Hospital Anxiety and Depression Scale, and EQ-5D-5L.

Results: Fourteen participants were randomised to receive Hybrid CBT, 11 to receive the self-help control treatment. Of the 14 in the Hybrid CBT group, 9 (64%) completed all four treatment sessions (4 discontinued due to poor health; 1 due to time constraints). Adherence to the self-help control treatment was not monitored. The total number of participants completing the 12-week and 24-week follow-ups were 12 (6 in each group; Hybrid CBT: 43%; Self-Help: 55%) and 10 (5 in each group; Hybrid CBT: 36%; Self-help: 45%). Based on the data available, candidate outcome measures appeared to be sensitive to changes associated with interventions. Thematic analysis of pre-post intervention interview data revealed satisfaction with treatment content among those who completed the Hybrid CBT whereas those in the Self-help control treatment wanted more contact hours and therapist guidance. Other practical suggestions for improvement included shortening the duration of each treatment session, reducing the amount of assessment paperwork, and minimising the burden of sleep and pain monitoring.

Conclusion: Important lessons were learnt with regards to the infrastructure required to achieve better patient adherence and retention. Based on the qualitative feedback provided by a subset of treatment completers, future trials should also consider lowering the intensity of treatment and streamlining the data collection procedure.

For peer review only - http://bmjopen.bmj.com/site/about/guidelines.xhtml

Trial registration: ISRCTN 17294365

Strengths and limitations of this study

- First feasibility trial in the UK to evaluate a new, brief but intensive Hybrid CBT for pain-related insomnia compared to self-help control in primary care.
- The Hybrid CBT was manualised, the delivery of which was supported by a comprehensive therapist training programme.
- Patient recruitment was tested in three different health centres of different demographic compositions and different socio-economic backgrounds.
- The mixed-methods approach provided both quantitative and qualitative information to inform the design and planning of a definitive trial.
- Rates of attrition and loss to follow-up were high in both arms.

CONSORT Checklist attached

CORE-Q Checklist attached

For peer review only

Introduction

Chronic pain is a major burden to primary care, accounting for five million GP appointments each year in the UK (1, 2). These pain patients usually present with multiple symptoms, with insomnia being one of the most common and disruptive comorbidities (2-4). In hospital pain clinics, as many as 90% of the patients report insomnia of a severity that warrants clinical attention (3, 5-8).

Conventionally sleep disturbance is seen as a secondary symptom to pain, but recent research has shown that poor sleep is actually a key driver for persistent pain and its associated distress and disability (9-13). Additionally, untreated insomnia is a significant risk factor for adverse health outcomes, e.g., hypertension, cardiovascular disease, obesity, diabetes, respiratory diseases and even increased mortality(14-20).

Although better sleep has long been emphasised by pain patients as an important treatment outcome (21, 22), insomnia is rarely a focus in pain management programmes. In primary care where most chronic pain patients are managed, hypnotics continue to be first-line treatments for insomnia despite the limited evidence supporting their long-term efficacy and safety (23). Their prolonged use can result in undesirable side effects, increasing the risks of falls, road traffic accidents, dementia and mortality in the long term, particularly in older adults (24-28). The risks multiply when the effect of polypharmacy is factored in. The combined use of benzodiazepines and opioids produce significant respiratory depression and is thought to contribute to the recent sharp rise in unintentional prescription drug overdose deaths (29).

Psychological interventions offer a promising treatment alternative. The efficacy of cognitive behaviour therapy for primary insomnia (CBT-I) has been demonstrated by multiple systematic reviews and meta-analyses (30-36). However, these treatments are often not available for chronic pain patients because of the lack of an empirically-validated treatment protocol adapted and tailored for this population (37), a shortage of skilled therapists (38-40), and an absence of essential infrastructure for CBT delivery in primary care (28, 30, 41).

For peer review only - http://bmjopen.bmj.com/site/about/guidelines.xhtml

We have recently evaluated a talking therapy specifically modified for patients with pain-related insomnia (42). The intervention simultaneously tackled chronic pain and insomnia, combining select components of CBT-I with interventions targeting the cognitive-behavioural processes maintaining chronic pain. The Hybrid CBT was delivered as an individual therapy over four weeks through weekly two-hour sessions. The treatment dosage was eight hours in total, approximating the optimal dose recommended for CBT for insomnia disorders (43) within a stepped care model (39). In our pilot study with patients recruited from hospital pain clinics (i.e., secondary care), the Hybrid CBT was associated with greater improvement in sleep at post-treatment compared with a symptom-monitoring control procedure (treatment effect size of Hybrid CBT: $d_H = 2.92$; control: $d_C = 0.56$)(42). Pain intensity did not change ($d_H = -0.13$; $d_C = 0.14$), but the Hybrid CBT was associated with greater reductions in pain interference ($d_H = 1.92$; $d_C = 1.19$), fatigue ($d_H = 1.81$; $d_C = 0.15$) and depression ($d_H = 0.94$; $d_C = -0.04$) than control (42).

The current study tested the feasibility of adapting and implementing the Hybrid CBT in primary care, using a mixed-methods approach. With a small patient sample across three primary care centres from localities with different social-economic and demographic characteristics, our overarching aim was to generate information to inform the development of a definitive randomised controlled trial (RCT) for evaluating the clinical outcomes and cost-effectiveness of the Hybrid CBT within the UK National Health Service (NHS) setting. The focus of the current study was therefore not on detecting differences in outcomes between the Hybrid CBT and the control groups, but on evaluating the technical and logistic feasibility of a full-scale study. Specifically, our aims were to: (i) check participant's willingness to be randomised between the Hybrid CBT and self-help control intervention, (ii) assess recruitment strategies of practices, staff and patients, (iii) estimate attrition rates throughout the study, (iv) evaluate performance and acceptability of candidate outcome measures, and (v) evaluate the data collection method. Hence, in this article, we report the methods and findings from the feasibility trial, along with qualitative findings based on our process evaluation of patient experience.

For peer review only - http://bmjopen.bmj.com/site/about/guidelines.xhtml

Methods

Patient and public involvement (PPI)

Two patient representatives with prior training for research involvement were recruited for this study through the Warwick Universities/User Teaching and Research Action Partnership. Our PPI representatives were co-applicants of the grant application and members of the project management committee. They were involved in most aspects of the study including trial design, therapist training, trial implementation and results discussion. Additionally, a member of our research team with a chronic pain condition gave significant insights into the running of the trial on top of their technical research expertise.

Trial setting and design

The feasibility study was a randomised controlled trial with a multi-centre, parallel-group design situated within primary care. Treatments were offered to adults living with chronic pain and insomnia in the community and delivered by trained health psychologists at the primary care centre from which the patients were recruited. (ISRCTN registration number: 17294365)

Participants were randomly assigned to receive one of two trial treatments for pain-related insomnia, in addition to treatment as usual (TAU). Here, TAU referred to the existing advice and prescribed medications for pain and insomnia that the participants were receiving. The assumption of TAU reflected the clinical reality that most patients with these chronic conditions would have already received some medical advice or treatment for their symptoms.

Treatments

Hybrid CBT

The Hybrid CBT comprised select components of CBT-I and interventions designed to target cognitive-behavioural processes maintaining chronic pain. The core components of the treatment were described in Tang et al. (42) and included sleep psychoeducation, stimulus control therapy, sleep restriction therapy and cognitive therapy for addressing insomnia-related cognitions and behaviours

For peer review only - http://bmjopen.bmj.com/site/about/guidelines.xhtml

common amongst chronic pain patients. It also included individual formulation, goal setting and behavioural activation, components for reducing pain catastrophising and safety-seeking behavior, and reversing mental defeat for the management of chronic pain. The treatment was manualised for this study to facilitate therapist training; the guiding treatment principles were laid out in the treatment manual to support flexible treatment delivery for this patient group with complex needs.

The format of the treatment can be described as 'brief, intensive, and concentrated' (44). Each patient allocated to the Hybrid CBT group was offered a total of four individual sessions on a weekly basis. Each session was approximately two hours long. The idea was to maintain the level of treatment content whilst minimising the burden of travel and duration of treatment, which may hinder treatment engagement in this patient group (45).

Self-help control treatment

Existing patient reading materials were amalgamated (with minimal modification) into four booklets to provide a self-help treatment on managing chronic pain and insomnia. The materials on insomnia were collated from the self-help treatment developed by Morgan et al. (46) Compared to treatment as usual, the use of these self-help materials was found to be effective in improving insomnia symptoms in older adults attending primary care for sleep and other comorbid chronic conditions (post-treatment effect size on sleep measures ranged from d = 0.69 – 0.7) (46). The self-help booklets were posted to the patients' homes, one at a time on a weekly basis. The content of the self-help gave equal coverage on chronic pain and insomnia management, approximating the structure and content of the Hybrid CBT. The self-help control treatment represented an active treatment control minus therapist contact.

Therapists

In the UK, clinical psychologists are not usually a part of the primary care medical team. Patients are often referred to see a psychologist for psychological interventions on an as-needed basis. Other provisions of care exist, e.g., in-house

For peer review only - http://bmjopen.bmj.com/site/about/guidelines.xhtml

counsellors and local Improving Access to Psychological Therapy (IAPT) teams, but availability of these services varies depending on locality and resource allocation. Previous trials of CBT-I in primary care have recruited nurse/health visitors for the delivery of treatment (47). Given the content (i.e., treatment of two complex health conditions), approach (non-protocol-based CBT), format (individual, brief but intensive) and focus (behaviour change) of the Hybrid CBT, health psychologists were chosen as therapists for this trial.

Following targeted recruitment via health psychology training centres and professional networks across the UK, six health psychologists - fully qualified or in the latter stages of their stage II doctorate – were selected to receive three days of intensive training offered by the team. Three withdrew before the trial commenced due to clashes with existing employment/study commitments (e.g., maintaining private practice and completing other training) and the distance of travelling involved in the process of treatment delivery (e.g., from London or Staffordshire, where the recruited therapists were based, to Coventry, Rugby and Warwickshire, where the primary care centres were located). The remaining three (100% female) went on to become the trial's therapists, which involved further training at their own pace via learning resources posted online, case piloting, regular individual supervision by experienced health psychologists on the team (HS, SP, NKYT), and travelling across sites to offer treatment to the patients in their localities.

Patients

We recruited 25 people living with chronic pain, between April 2016 and April 2017 from three primary care centres in Coventry/Warwickshire of different demographic compositions (respectively having 2.1%, 3.7% and 25.8% non-white population) and different social-economic backgrounds (respectively scoring 1, 5 and 8 on the 1-10 Index of Multiple Deprivation) (48).

We identified participants for the study from the electronic registers held by the participating centres. Patients were initially screened by searching each centre's

For peer review only - http://bmjopen.bmj.com/site/about/guidelines.xhtml

electronic patient records for inclusion criteria. We did two searches, with search terms broadened to include specific medications for the second search.

Inclusion criteria were individuals (i) aged 18 years or above, (ii) English-speaking, (iii) registered with one of the participating centres, (iv) with a history of chronic pain and insomnia (as indicated by their medical records), with (v) pain of at least moderate severity (≥4/10 on a present pain intensity numerical rating scale for at least 6 months), and (vi) clinical insomnia (≥15 on the Insomnia Severity Index, ≥3 nights a week, ≥1 month in duration). Criteria vi mapped onto the American Academy of Sleep Medicine Research Diagnostic Criteria for Insomnia Disorder (49), which is consistent with the DSM-5 diagnostic criteria although the latter adopted a three-month duration criteria (50).

Participants taking pain/sleep medications on a stable regimen were included if they met criteria of the study. However, we excluded potential participants with diagnosed or suspected medical/psychiatric/sleep disorders for which CBT-I was contraindicated as first-line treatment or those who had recently enrolled in or were completing a pain management programme, or other psychological treatments for pain or sleep.

As this was a feasibility study, no formal power calculation to test the effectiveness of the intervention was possible. The current sample size was determined by practicalities and considerations that it was sufficiently large to ensure that randomisation was acceptable and to buffer against atypical attrition.

A randomisation list was created by the trial statistician (HP) using random blocks of varying sizes (block length=4 or 6). Blocks were generated in groups of patients at a 1:1 ratio, and stratified by centre from which the participants were recruited. Patients were randomised sequentially as they became eligible for inclusion in the study. Allocation was concealed using 'e-envelopes', which were macro-enabled MS Excel files preserved as read-only with automatic saving on any alteration to the file to give an audit log. This method was considered cost-efficient for a small-scale feasibility study. Furthermore, the study statistician only released these e-

For peer review only - http://bmjopen.bmj.com/site/about/guidelines.xhtml

envelopes and their passwords in small batches on request by the trial coordinator (CM). The study statistician had no contact with participants at any point in the study.

Quantitative outcome measures

We piloted the use of five validated questionnaires to collect data in this population. Our candidate primary outcomes were the Insomnia Severity Index (ISI) (51) and Brief Pain Inventory (BPI) pain interference subscale (52). Candidate secondary outcomes included the present pain intensity rating from the BPI (52), Multidimensional Fatigue Inventory (MFI) general fatigue score (53), Hospital Anxiety and Depression Scale (HAD) (54) and EuroQoL EQ-5D-5L (55), for which health utilities were calculated using the UK tariff of the EQ-5D-5L value set (56). The health thermometer score was also reported.

Several process measures were included as part of the assessment to inform treatment and elucidate the role of hypothesised treatment mechanisms. These were the Pain Catastrophizing Scale (57), Pain Self-Perception Scale (58) Anxiety and Preoccupation about Sleep Questionnaire (59), Dysfunctional Belief and Attitude about Sleep Scale–16 (60), and Pain-specific Dysfunctional Belief and Attitude about Sleep Scale (61).

In addition, the participants were asked to complete a daily sleep diary modified from the Consensus Sleep Diary (62) and to wear an actigraph (Model: MW8, supplied by CamNTech) for a week for baseline and 12-week follow up assessments to examine the feasibility of incorporating objective sleep measures for future trials.

Statistical analysis

As the aim of the current trial was to evaluate study feasibility, not treatment efficacy, no formal between-group analyses were planned. However, planned analyses consisted of the generation of descriptive statistics for all time points, across all participants as a group, and the Cronbach's alpha of the candidate primary outcome measures to assess if internal consistency of these measures

For peer review only - http://bmjopen.bmj.com/site/about/guidelines.xhtml

was adequate when administered in a chronic pain patient sample with a minor modification (i.e., the sleep item was removed from the BPI to avoid criterion contamination). The candidate primary outcome measures were also checked for correlation to establish if co-primary outcomes were needed for a definitive study.

Qualitative participant interviews

Face-to-face semi-structured interviews were conducted before and after the intervention to explore participants' expectations of the intervention and their overall experience post-intervention. These interviews were carried out individually with a subgroup (20%) of participants enrolled in the trial by a Research Fellow who specialises in qualitative health service research but was not involved in the treatment design and delivery process (VPN). The interviewer had no prior contact with the interviewees. Seed questions used to prompt these conversations are shown in Box 1.

Box 1. Seed questions used in the semi-structured interviews pre- and post-intervention for both Hybrid CBT group and Self-help control group arms.

**A. Pre-intervention interview**	
1.	We are very interested in your journey. When did you first notice your pain-related sle[illegible] disturbance?
2.	Did you put it down to anything?
3.	Did you talk to anyone about it?
4.	What did you do to try to help?
5.	Does anything make it better or worse?
6.	Have you seen any health professionals or other practitioners?
7.	What do you think is going to happen in this study?
8.	What are you hoping to get from this study?

**B. Post-intervention interview**	
1.	How did you get on with the study?
2.	Did you get any benefit from the study (attending the sessions/receiving the booklets)?
3.	What worked for you?
4.	What did not work for you?
5.	Would you recommend this type of programme to other people?
6.	If we ran this programme in a larger study is there anything you would change?
7.	How did you get on with the paperwork?
8.	How did you find the process of being in a research study?

For peer review only - http://bmjopen.bmj.com/site/about/guidelines.xhtml

Timelines of the participant's symptom and treatment journey were drawn during the interview and field notes were written immediately post interview to promote reflexivity. The interviews were held at the participant's primary care centre, and lasted for about 60 minutes each. Interviews were audio-recorded on an encrypted digital device. Recordings were transcribed verbatim and anonymised. Data transcription was supported by another doctoral-level Research Fellow with experience in qualitative health care research but again not involved in the study design and treatment delivery process (VEJC). NVivo software was used for managing the data for analysis of the interview transcripts.

Thematic analysis was performed on the transcripts by the CI (NKYT) following the six key processes recommended by Braun and Clarke (63); familiarisation with the data set, generating initial codes, searching for themes, reviewing themes, defining and naming themes, and report production. Codes and themes extracted were then reviewed by the interviewer (VPN) and a second experienced qualitative researcher on the team (DE) to check for accuracy. Comments received were then used to revise the analysis. Themes were extracted with the awareness that questions asked in the pre-intervention interviews were different from the post-intervention interview, and hence the analysis avoided referring to any within-participant change across these interviews. Constant comparisons were applied when analysing the post-intervention interviews, contrasting the experience of the participants assigned to receive Hybrid CBT with those assigned to the Self-help control treatment.

Results

Search results

The searches identified a total of 1,434 potentially eligible patients across the three primary care centres (9.8% of all records). After receiving an invitation to become part of the study, 85 patients responded and were invited to telephone (n=45) and then in-person (n=40) screening to be further assessed for eligibility. Of the 25 (55.6% of screened) participants who were found to be eligible, all were recruited into the study. This corresponded to a pick-up rate of 1.7 per 1000 registered patients and a successful recruitment rate of 1.9 participants per

For peer review only - http://bmjopen.bmj.com/site/about/guidelines.xhtml

month, or 0.6 participants per centre per month. Figure 1 specified the number and reasons for exclusion at screening and subsequent stages.

Baseline participant characteristics

Table 1 summarises the socio-demographic details provided by the study participants at baseline (n=25). At the group level, 56% of the participants were female with an average age of 49 years and a mean BMI of 29. The majority of the participants were white (96%) and married or living as married (60%). Forty-four percent of the participants had secondary level education as their highest educational qualification. At the time of the study, 40% of the participants were in paid work whilst 60% were unemployed, retired or engaged in other forms of activity, with 36% receiving some form of social benefits.

The mean reported duration of pain was 11 years. Most of the participants described their pain as "constant" and the median number of pain sites reported was 5, with lower back as the most commonly identified pain site, followed by neck, shoulders, joints, legs, knees, arms, upper back, head and abdomen. The baseline present pain intensity VAS was 6.1, pain interference score 6.6, and ISI 18.9. Treatment adherence and attrition

All 14 participants assigned to the Hybrid CBT group completed session 1, nine completed (64.3%) sessions 2, 3, and 4. Of the five participants who discontinued, four gave 'poor' health and one gave 'lack of time' as reasons for withdrawal (Figure 1). Those withdrawn were recruited from primary care centres with more severe deprivation indices. For the self-help control group, all leaflets were mailed to the participants weekly, as per protocol. No further adherence data were collected. At 12-week, six participants were successfully followed up in each arm; at 24-week five. This gave an overall participant retention rate of 48% at 12-week and 36% at 24-week.

For peer review only - http://bmjopen.bmj.com/site/about/guidelines.xhtml

Table 1. Participants characteristics as measured at baseline.

**Baseline variable**		**All**<br><br>**N=25**
Recruitment centre (n, %)	Primary Care Centre 1	8 (32)
	Primary Care Centre 2	10 (40)
	Primary Care Centre 3	7 (28)
Gender (n, %)	Female	14 (56.0)
Age: years (mean, SD)		49.3 (9.8) †
BMI: kg/m^2 (mean, SD)		29.3 (7.6)
Ethnicity (n, %)	White	24 (96)
Relationship status (n, %)	Cohabiting/ Married/Engaged	15 (60)
	Single/ Separating	10 (40)
Education (n, %)	No formal qualifications	8 (32)
	Secondary	11 (44)
	Degree/professional qualification	6 (24)
Employment (n, %)	Paid work	10 (40)
	Retired/medically retired	6 (24)
	Unemployed	5 (20)
	Other	4 (16)
Receiving benefits? (n, %)	Yes	9 (36) †
How long have you had the pain (minimum, in years) (mean, sd)		11.0 (9.0) ‡
What the pain is like (n, %)	Constant	20 (80)
	Recurrent	3 (12)
	Occasional	1 (4)
	Missing	1 (4)
No. of painful places given (median, range)		5 (1 – 8)
Where is the pain (n, %)	Head	3 (12)
	Neck	17 (68)
	Shoulders	18 (68)
	Upper Back	6 (24)
	Lower back	18 (72)
	Arms	9 (36)
	Legs	15 (60)
	Knees	13 (52)
	Abdomen	2 (8)
	Joints	15 (60)
	Other	7 (28)
Brief Pain Inventory (mean, sd)	Current pain severity	6.1 (1.5)
	Current pain interference	6.6 (1.5)
Insomnia Severity Index (mean, sd)	Total score	20.1 (4.9)

† 1 participant missing data. ‡ 3 participants missing data.

For peer review only

Table 2. Descriptive statistics of the candidate primary and secondary outcome measures, as well as process measures.

	Baseline		12 weeks		24 weeks	
	All (n=25)	No. of valid response (n, %)	All (n=25)	No. of valid response (n, %)	All (n=25)	No. of valid response (n, %)
Primary outcomes						
ISI (mean, sd)	20.2 (4.7)	24 (96)	14.4 (10.3)	10 (40)	14.8 (11.8)	8 (32)
BPI interference	6.1 (1.7)	24 (96)	4.9 (2.5)	12 (48)	4.7 (2.7)	8 (32)
Secondary outcomes						
BPI - pain intensity	6.2 (1.6)	24 (96)	5.1 (2.4)	12 (48)	5.5 (2.6)	8 (32)
MFI - general fatigue	16.3 (3.6)	24 (96)	13.8 (3.7)	12 (48)	14.9 (3.2)	8 (32)
HAD - anxiety	9.7 (3.0)	24 (96)	6.7 (5.5)	12 (48)	8.9 (4.9)	8 (32)
HAD - depression	8.4 (3.5)	24 (96)	7.3 (4.6)	12 (48)	7.8 (5.1)	8 (32)
EQ-5D - health thermometer score	50 (17.5)	24 (96)	58.9 (15.2)	12 (48)	58.8 (23.0)	8 (32)
EQ-5D - utility score	0.60 (0.19)	24 (96)	0.57 (0.29)	12 (48)	0.56 (0.33)	8 (32)
Process measures						
PCS - pain catastrophising	15.7 (9.2)	24 (96)	9.5 (8.5)	12 (48)	10 (8.6)	8 (32)
PSPS - mental defeat	30.8 (23.6)	23 (92)	21.9 (27.3)	12 (48)	14.7 (21)	6 (24)
APSQ - sleep anxiety	68 (21.7)	24 (96)	50.2 (32.4)	12 (48)	49.2 (37)	9 (36)
DBAS - sleep beliefs	5.6 (2.1)	21 (84)	4.1 (2.9)	10 (40)	4.0 (3.3)	8 (32)
PBAS –-pain-related sleep beliefs	7.1 (2.1)	21 (84)	5.1 (3.4)	12 (48)	5.0 (3.7)	9 (36)

Notes. ISI = Insomnia Severity Index. BPI = Brief Pain Inventory. MFI = Multidimensional Fatigue Inventory. HAD = Hospital Anxiety and Depression Scale. EQ-5D = EuroQol EQ-5D-5L. PCS = Pain Catastrophizing Scale. PSPS = Pain Self Perception Scale. APSQ = Anxiety and Preoccupation about Sleep Questionnaire. DBAS = Dysfunctional Beliefs and Attitudes and Sleep scale. PBAS = Pain-related Dysfunctional Beliefs and Attitudes about Sleep scale.

For peer review only - http://bmjopen.bmj.com/site/about/guidelines.xhtml

Outcome measures

Both primary outcome measures appeared to have excellent internal consistency. The Cronbach's alpha for the cohort was 0.94 (95% CI 0.92-0.97) for the ISI and 0.88 (95% CI 0.87-0.95) for the pain interference score. Even though the BPI| sleep item was dropped to avoid criterion contamination, there was a strong non-zero correlation between ISI and BPI interference score (r = 0.81, 95% CI 0.67-0.89).

Descriptive statistics of all primary and secondary outcome measures, as well as process measures, by assessment time points, are shown in Table 2. No adverse events were reported in either allocation groups.

Participant Interviews

Five patients were interviewed; three were from the Hybrid CBT group and two were from the Self-help control group. All completed both pre- and post-intervention interviews, except one in the Hybrid CBT group who only completed the pre-intervention interview.

Pre-intervention

Discussions in the pre-intervention interviews gave rise to six interesting themes, capturing several prominent psychosocial characteristics of the participants enrolled in the feasibility study. These themes (presented in Table 3 with additional illustrative quotes) were concerned with the participants' sense of identity, personal adversities, treatment experience and coping strategies, perceived pain-sleep relationship, satisfaction with current service, and treatment expectations.

Pain changed who I am

There was a sense of damaged identity shared across the five participants interviewed. They appeared to define themselves by their experience of pain and losses due to pain. Frequent comparisons were made of what life was in the past, with what life is now and what life should be like at a certain age. The way in which participants spoke of their struggle with pain carried a sense of mental defeat.

> *"I've been suffering with sleep deprivation for many years...pain the same. ... it destroyed everything really... life changed a hell of a lot, ... it stole my life away." (Patient C)*

For peer review only - http://bmjopen.bmj.com/site/about/guidelines.xhtml

Table 3. A summary of findings from the pre-intervention interviews, with additional quotes and implications for future trial planning.

Theme	Additional example quotes and/or additional notes* (Patient code for example quotes e.g., *Patient X*)	Implications for future trial planning
Pain changed who I am	*"…you're in pain the whole time… [you] can't move and somebody's got to help you out of bed, which really at 48 I shouldn't be like that. … I feel I'm always going to be a person with pain…"* (Patient D)<br><br>*"I like to think that I'm quite strong but equally I feel that…I've given up. I'm frustrated [because] I'm hurting. I can't escape."* (Patient E)	Patients' damaged sense of identity - and the related psychological processes that feed into it - should be kept as a core target of the hybrid treatment and measured for pre-post intervention changes.
Pain and sleep did not occur in psychosocial vacuum	* *No one single quote could satisfactorily illustrate the complexity of the psychosocial contexts described by the participants, and without risks of revealing their identities.*<br><br>*Example adversities cited included ill health, mental health problems, car or work accidents, assaults, relationship breakdown, problems experienced by dependents or close family members, being a carer, job redundancy/ unemployment, financial difficulties, homelessness, and bereavement.*	Whilst the current hybrid treatment has room to support flexible treatment delivery for patients with complex needs, more considerations should be given to the context in which the treatment is being delivered, as well as to practical support required to enable the most disadvantaged/burdened patients to access treatment.
Participants were not treatment naïve	*"You just try to help yourself a little bit but, whether that's a good or bad thing I don't know."* (Patient A)<br><br>*"I'm trying to think myself healthy…I've tried …books … having your room right and spraying your pillow … all sorts of things…"* (Patient D)<br><br>*"…there wasn't nothing that I haven't already seen or read or something before…."* (Patient E)	Self-help treatments may not be considered as a satisfactory treatment option by this non-treatment naïve clinical population.<br><br>An active alternative treatment with therapist contact may be a more appropriate control intervention in future trials.

For peer review only - http://bmjopen.bmj.com/site/about/guidelines.xhtml

Pain was thought to be the primary cause of sleep problems	*"I'm not just gonna blame the pain…I've got a [teenage] son who's causing … I'm not naïve to think that's not a contributing factor [to sleep problems]… And I do stress … that's just in my nature."* (Patient D)<br><br>*"[when] I wasn't sleeping the pain seemed more unbearable… Unbearable, [because] I was tired… And I felt run down it just seemed worse I think…"* (Patient E)	If patients hold a rigid belief that sleep will never improve unless pain is resolved, it would be difficult to get their buy-in to the Hybrid CBT on offer. As such, these beliefs need to be addressed upfront in the information sheet or during recruitment, to improve treatment uptake and subsequent adherence.
Participants were dissatisfied with the services available	*"Most doctors these days don't…give the time of day. They've got your prescription written out before you go in."* (Patient C)<br><br>*"[Interviewer: So have you talked to anybody about your pain and sleep?] Only my GP…And they sort of got painkillers. They don't really like to give sleeping tablets anymore. Um... They advised over the counter ones...which work to an extent …."* (Patient B)<br><br>*"I don't feel that this surgery offers a lot of [non-drug treatments]....it can give (medication), but obviously I've stopped taking all tablets now for five weeks and I can't see if there's a difference from taking tablets to the placebo effect of fear that at least by reaching for the tablet there was something to help me."* (Patient E)	The issue of validation (or the lack thereof) is not unique to chronic pain patients, but highlights the importance for future trials to provide generic clinical skills training to the study therapists (health psychologists in the current study, or other suitably trained allied health care professionals with appropriate expertise in future trials). This will allow the provision of quality therapist contact, which is valued by our target patient group.
Participants' treatment expectations were high	*"…just to help control pain and sleeping…you can't work miracles but it might be something that can help me…To be honest … I'm hoping … you might have the magic cure, you never know."* (Patient A)<br><br>*"A bit more sleep. More than anything. I find if I'm tired, … the pain seems worse or I'm just not able to cope with it as well…So my theory is if I can just get a bit more sleep I can perhaps cope better with the pain."* (Patient B)	Proactive management of patients' treatment expectations at the outset of treatment, or as early as the enrolment stage in future trials, may help minimise attrition and unnecessary demoralisation.

For peer review only - http://bmjopen.bmj.com/site/about/guidelines.xhtml

Pain and sleep did not occur in psychosocial vacuum

The interviewees' descriptions of their treatment journeys revealed that the issues of chronic pain and insomnia were embedded within a larger context of personal adversities. It was difficult to tell whether these were psychosocial triggers or consequences of chronic pain and insomnia. Specifics of these adversities revealed themselves at different places of the interviews, where details of personal lives were volunteered to situate the conversation. Adversities came not in isolation but in clusters as chronic pain and insomnia became increasingly more severe and disabling. Example adversities cited included ill health, mental health problems, car or work accidents, assaults, relationship breakdown, problems experienced by dependents or close family members, being a carer, job redundancy/unemployment, financial difficulties, homelessness, and bereavement.

Participants were not treatment naïve

Perhaps an artefact of self-selection bias in an RCT, all participants interviewed had tried to manage their pain and insomnia using a combination of drug and non-drug strategies, invariably with limited success. The self-helping spirit is a double-edge sword. If the right treatment is identified, it could facilitate engagement and maximise treatment gains. It could also drive people to take bold steps to keep pain and sleep problems under control, using strategies that are not necessarily recommended by current evidence-based guidelines, which may lead to dashed hope and further demoralisation.

> *"…Now I try to keep [pain medication] in my system all the time so there's always something there …rather than waiting for the pain to start and then taking [the medication]. [Interviewer: Right so have you done that on the advice of somebody or off your own back?] No just off my own back…" (Patient B)*

Pain was thought to be the primary cause to sleep problems

All participants interviewed shared a strong belief that pain was a major cause of their sleep problems. However, there was the awareness that other factors might also play a role in aggravating the sleep problems and that not sleeping well could have a reciprocal effect on the pain.

> *" [It's the pain that] keeps me awake…As soon as you have pain then wakes…Sometimes you can't sleep because of the pain, and then you're up all night, you get yourself angry with yourself…" (Patient C)*

For peer review only - http://bmjopen.bmj.com/site/about/guidelines.xhtml

Participants were dissatisfied with the services available

All participants interviewed have had much interaction with multiple health service providers. They were most frustrated when they felt they were not being listened to or misunderstood by their GPs. They were also not happy with a lack of effective treatment choices, noting a contradictory combination of a heavy reliance on drugs for pain control with a general reluctance to prescribe sleeping tablets for insomnia.

> *"I kept going with all this pain problems and that, never get nowhere… Just felt as though it was all in my head, nobody would listen to me…" (Patient A)*

For peer review only

Participants' treatment expectations were high

All participants interviewed showed understanding that the interventions offered were not 'magic cures', but nonetheless had high expectations for the treatments, reflecting the constant tension between ideally what the patients want ("no pain" and "lots of sleep") and realistically what can be offered by an intervention designed to optimise management of these problems.

> *"Obviously it's not going to be magical overnight but even if it could give me ideas if I'm in that position how I should be reacting …. Well my future…hopefully I will have no pain … lots of sleep and no stress." (Patient D)*

These themes, together, contextualised the feasibility study, offering finer insights into the life circumstances of those who actually signed up for pain-related insomnia treatment.

Post-intervention

Discussions in post-intervention interviews revealed aspects of the intervention liked and disliked by the two interviewees (as graphically summarised for the Hybrid CBT in Figure 2). Analysis of these factors was carried out separately for the Hybrid CBT and the Self-help control groups, to generate clear suggestions as to how each of these interventions could be tweaked and improved from the patient experience perspective.

Hybrid CBT

Besides "very good practical advice" on sleep, both interviewees who completed the Hybrid CBT appeared to most appreciate the intervention for giving them a new understanding of sleep as well as themselves. In particular, they seemed to like the fact that these new insights enabled them to improve their sleep patterns and to change the

For peer review only - http://bmjopen.bmj.com/site/about/guidelines.xhtml

way they think about and react to situations in life. Their success in improving sleep also appeared to have boosted their confidence in initiating changes in other areas of their lives; a spill-over therapeutic effect.

> *"... You know, understanding how you sleep really... a lot better frame of mind now and a lot better myself you know what I mean? ...Because they're slowly dispersing these worries are, and things like that, a lot to look forward to, and different things, more positive now… [It]changes the way you think a little bit and me talking to [the therapist] that I want to walk more and do more... It makes you think like… I can do something about it, I've done this, I've done um this sleep pattern and that…" (Patient A)*

> *"I'm remembering everything and I really liked the study 'cause it helps me do things different… it's all about retrain(ing) your brain to you know understand our sleep patterns and things we do in our life, what we do and it works it really really works…" (Patient C)*

Both participants interviewed were also very positive about the one-to-one, face-to-face interaction they had with the therapists. They felt that they were being listened to, and really appreciated the fact that the therapists went to the community to offer the intervention in their locality. They also felt that they managed to derive a greater understanding of the treatment materials because they could talk to someone and ask questions as they arose, compared to reading the information on their own. Further, they felt that being able to talk confidentially to someone knowledgeable about their symptoms and experience had allowed them to process difficult emotions that may or may not be directly linked to the experience chronic pain.

> *"...the one-to-one sessions are good... They are really good. Um I don't know how it would work in a group." (Patient C)*

> *"...I think you understand it a bit more by talking [to] somebody" (Patient A)*

> *"... I'd go home have a good read about [the treatment materials], some of it I didn't understand but then when I come back I'd ask… or she'd explain to me… write it down and show me that this is the way to do it... So I was learning a new skill… I don't think I'd have took it so serious ... I don't think I would've, just read a couple of leaflets and oh yeah and then popped it down the side, I wouldn't have thought about it…" (Patient C)*

> *"And at least they could come to me and I didn't have to get there. I wouldn't have done that you see?... Cause it's on your door step it's a lot easier ... and you fit it in" (Patient A)*

Three issues of the Hybrid CBT required attention. First, although sleep monitoring was an essential component of the intervention, the participants interviewed found

For peer review only - http://bmjopen.bmj.com/site/about/guidelines.xhtml

completing the sleep diary tedious and possibly sleep-interfering as they felt obliged to clock their activities and remember everything they had done. Second, the participants found components of the sleep restriction and stimulus control therapy hard to follow. Although their sleep became more consolidated as a result of these treatment components, they did not particularly enjoy the experience and felt it was important to be able to personalise the intervention. Third, the participants struggled to apply the sleep restriction therapy during pain flare ups. Further adjustments to the pace and method of the therapy may be required considering that an increase in pain is a possible side effect during the initial stage of the sleep restriction therapy. Application of digital technology may also help reduce the burden of data collection.

> *"[The monitoring/diary] was a bit monotonous, but then they're not going to know unless you write it down..." (Patient A)*

> *"I didn't like that side of it (the sleep restriction and stimulus control therapy) 'cause I do like to stay in bed..." (Patient C)*

> *"...but then you can have a flare up and then you're struggling...you know?" (Patient A)*

> *"... I was following it all um you know we've come to like an agreement on timing to go to sleep and timing to get up..., but the problem is I've got a bad neck"..."It's really sore and moving it sometimes I can't move it ... Can't go out, can't drive, can't do anything cause my necks playing me up, so it knocked my sleep pattern a bit out of proportion..." (Patient C)*

Self-help control

The Self-help control intervention was chosen to represent what was the best treatment option available for patients with chronic pain and insomnia in primary care. However, few positives were said about the Self-help control intervention, except that the advice given was 'sound' and 'clear'. Whilst both participants interviewed felt that overall the intervention was helpful, they felt that they were not learning much new information and could not pinpoint any specifics as to why and how the intervention was helpful. Their memories of what was being discussed in the self-help booklets were also very vague. They felt that the information provided in the booklets overlapped with information available in existing self-help books or internet sources. They also indicated that they wanted more contacts with health care professionals rather than being left to their own devices.

For peer review only - http://bmjopen.bmj.com/site/about/guidelines.xhtml

> *"Yeah the study itself is alright…"… "On the sleep [side], the things it gives are clear and can be helpful, but to me I already practiced those things anyway… but I've already read up on lots of things and done, so there wasn't a lot there that was explaining that I could take away and thought I've absorbed something new."" (Patient E)*

> *"So something's obviously helped along the way but I can't put my finger on one exact thing." "[Interviewer: is there anything, is there anyway we could improve it do you think?] In a way perhaps a bit more contact because obviously you have the initial study ... I saw you for that interview sort of in the middle ... And then now, but other than the booklets then you are just sort of left to it.... There's no contact whatsoever.... So some contact would've been useful." (Patient B)*

Other feedback from participants assigned to both groups were concerned with the randomisation procedure, intervention format, and data collection method. On randomisation, the participants in the Self-help control group indicated that it "would have been nice to have a choice" whereas those in the Hybrid CBT group imagined that they "wouldn't have bothered" with the treatment or would have benefited less had they been assigned to the Self-help control group. There was also an agreement from participants of both groups on the intensity of the treatment, with too much to read and/or report. Whilst they did not have an issue with automated data collection devices (i.e., actigraphy), they had found it difficult to complete the sleep diary and recommended that the paper work of the treatment/study to be reduced.

Discussion

Delivering a brief but intensive intervention for the self-management of chronic pain and insomnia in primary care has proved to be challenging. Important lessons were learnt with regard to the infrastructure and trial design required to achieve better patient recruitment, treatment delivery, intervention adherence, and patient retention.

Were the patient identification and recruitment strategies viable?

Two searches were conducted to identify potential participants from electronic records. The initial search that used diagnoses as search terms identified only 263 potential participants across the three participating centres despite the high prevalence rates of both health conditions (64-67). The unusually low return might be explained by the fact that physician records did not always list sleep or chronic pain as diagnoses. A second search using a broadened set of search terms (including medication prescribed) was more successful, identifying 1,434 potential participants (inclusive of the 263 previously identified). Of those who responded to the invitations and were invited to full screening;

For peer review only - http://bmjopen.bmj.com/site/about/guidelines.xhtml

nearly a third were randomised into the study (63%). Of those who were not randomised, approximately equal numbers were ineligible and declined to attend full screening. This indicates that once found, recruiting patients into the study may not be an issue for a larger study, but capturing interested patients in the first place would be a challenge. Whilst the current pick-up rate of 1.7 per 1000 patients was satisfactory, future trials should multiply the number of recruiting primary care centres to ensure that the recruitment target will be reached in a timely fashion.

Previous research has found that patients with insomnia tend to trivialise their symptoms and did not seek treatment due to beliefs that one should be able to cope with insomnia alone (68). Despite the brief but intensive nature of the Hybrid CBT, four weekly sessions plus homework and data collection is a significant time commitment. Future trials should consider addressing these unhelpful beliefs during patient recruitment and incorporate an incentive system to motivate eligible participants to commit themselves to treatment. For example, a US trial of a six weekly 90-minutes group CBT for insomnia and pain among older adults with osteoarthritis offered a USD$2 cash incentive in the initial postal invitation (69). Participants in this study were paid volunteers. They received a USD$50 incentive payment after completing the baseline assessment and attending the first class (69). Similarly, an ongoing nurse-led brief insomnia treatment trial in the UK reimburses all participants after each completed follow-up visit; £5 at baseline, £10 at 3-month, £15 at 6-month, and £10 at 12-month (70). The current study offered the treatment for free but did not have the budget to incentivise enrolment and treatment attendance.

What were the rates of attrition?

Loss to follow up was high in this feasibility study, but return rates were generally higher in the Hybrid CBT group than the Self-help control group. Adherence to the Hybrid CBT appeared to be most vulnerable after session 1. Ill health was cited as the main reason for drop-out and incidentally, all patients withdrawn were recruited from centres with more severe deprivation indices. Those who managed to return to session two fully adhered to the rest of the treatment programme and appeared to report sizeable improvements across outcome measures. Future trials should seek to investigate whether any systematic attrition from the Hybrid CBT occurs following Session 1 compared to the control intervention, and if so, why? One size does not fit all; it would also be important for future trials to identify demographic, socio-economic, and clinical factors that predict treatment suitability, directly answering the ‘what works for whom’ question.

For peer review only - http://bmjopen.bmj.com/site/about/guidelines.xhtml

In addition to a dedicated budget for incentivising follow-up rates, practical support (e.g., travel reimbursement, appointment reminders, between-session technical support) may also be required to remove participation barriers and reduce attrition in future trials. Qualitative feedback from our participant interviews also indicates that streamlining the data collection with the help of digital technologies or the use of a more active control intervention with more patient contact may help buffer against attrition. Although the Hybrid CBT was generally well received by treatment completers, it could be simplified in future trials to reduce patient burden. We note that the majority of the treatment drop-outs coincided with the introduction of sleep restriction component of the therapy in session 1 and prior to their return to session 2, when they were expected to report on their progress. Considering that sleep restriction is the most difficult and counterintuitive component of the therapy to follow, future trials with additional support for commencing sleep restriction may achieve better adherence to the intervention.

How satisfactory and acceptable were the outcome measures and data collection methods?

Candidate outcome measures tested in the current trial appear to be psychometrically sound and have good face validity for the stated purpose of assessment. Both primary and secondary outcome measures showed changes in the direction anticipated for both arms of the trial over time. Care must, however, be taken when interpreting the positive responses that were reported due to biases associated with selective uptake and study attrition. We note that participants who remained in the study had lower levels of pain intensity at baseline than those who withdrew or were lost at the 12-week follow up (BPI severity: 5.9 v 6.4, not tested), although levels of pain interference and insomnia were more similar (BPI interference: 6.1 v 6.2, ISI: 20.3 v 20.1, not tested).

The pattern of change in the outcome measures was approximated by those of the process measures that assessed the hypothesised maintaining factors of pain and insomnia targeted by the treatments. Future trials should consider conducting appropriately designed analyses to examine whether changes in these processes mediate treatment outcomes. The combined use of a sleep diary and actigraphy is an important part of the assessment and treatment process (not reported in detail here), but the implementation of this recommended monitoring procedure proved to be challenging for the participants of the current study. Potential solutions to improve monitoring adherence in future trials may involve reducing the length of the monitoring procedure, more personalised data collection training and support, and the installation of analysis software in participating centres to minimise human errors in data transfer.

For peer review only - http://bmjopen.bmj.com/site/about/guidelines.xhtml

Strengths and limitations of this study

This was a small-scale feasibility study with a subgroup of participants being interviewed pre-post treatment for their experience participating in the trial. The specific aims were not focused on estimating treatment efficacy but on the implementation aspects of the running of the trial. We refrained from reporting the data by group or estimating effect sizes of treatment as the study was neither designed or powered to do so (71, 72) Bearing in mind the implementation issues discussed above, findings could be used to inform the design of any future definitive study but must be interpreted within its bounds of generalisability.

Therapist contact and the treatment focus on understanding how sleep works were highly valued by our interviewees. The delivery of the Hybrid CBT at the patient's doorstep was also positively received. Future trials should seek to maintain the quality of contact, although this will have to be supported by a robust health economic analysis that examines the cost-effectiveness of providing the Hybrid CBT in its current format as compared to in group settings or in the form of telemedicine or internet-based intervention.

Whilst no adverse event was formally reported, qualitative feedback suggested that increased pain – especially in the initial treatment period - may be a possible side effect of the sleep restriction component of the treatment. Further investigations into the frequency, timing, and severity of this potential side effect are required to ensure patient safety and, potentially, promote treatment adherence.

A previous meta-analysis has found the short-medium term outcomes of patients with comorbid or primary insomnia receiving bibliotherapeutic self-help were superior to those of waitlist control (73). Although self-help treatment is the best available non-pharmacological treatment options in many primary care settings, the trial team should consider whether it is an acceptable control intervention to offer in future trials. The post-treatment interviews revealed that our participants were not naive to self-help and appeared to be demoralised by the lack of therapist contact and fresh treatment content. Perhaps, a therapist-led educational intervention is a more fitting control for future trials evaluating the effectiveness of the Hybrid CBT in primary care.

For peer review only

Conclusion

The Hybrid CBT has the potential to fill an unmet clinical need. Through our feasibility trial, a treatment protocol and a corresponding therapist training programme have been developed to make the delivery of this brief but intensive intervention in primary care possible. In its current form, the Hybrid CBT may work for subgroups of individuals who manage to adhere to the programme. Future trials could overcome the challenges highlighted in this feasibility study by broadening recruitment catchment, incorporating an incentive system to motivate treatment uptake, streamlining the treatment to make it even more primary care friendly, and simplifying the data collection procedure to make it easier for the patients to take part and provide data for evaluation.

For peer review only

Figure legends

Figure 1. CONSORT flow diagram from screening (open boxes) to post-screening (filled boxes) processes in the study.

Figure 2. A summary of themes from post-intervention interviews of Hybrid CBT participants, highlighting the positives and negatives of the current treatment approach and content.

For peer review only

For peer review only - http://bmjopen.bmj.com/site/about/guidelines.xhtml

References

1. Belsey J. Primary care workload in the management of chronic pain. A retrospective cohort study using a GP database to identify resource implications for UK primary care. Journal of Medical Economics. 2002;5(1-4):39-50.
2. Ohayon MM. Relationship between chronic painful physical condition and insomnia. Journal of psychiatric research. 2005;39(2):151-9.
3. Tang NK, Wright KJ, Salkovskis PM. Prevalence and correlates of clinical insomnia co‐occurring with chronic back pain. Journal of sleep research. 2007;16(1):85-95.
4. Taylor DJ, Mallory LJ, Lichstein KL, Durrence HH, Riedel BW, Bush AJ. Comorbidity of chronic insomnia with medical problems. Sleep. 2007;30(2):213-8.
5. Bigatti SM, Hernandez AM, Cronan TA, Rand KL. Sleep disturbances in fibromyalgia syndrome: relationship to pain and depression. Arthritis Care & Research: Official Journal of the American College of Rheumatology. 2008;59(7):961-7.
6. Atkinson J, Ancoli-Israel S, Slater MA, Garfin SR, Gillin C. Subjective sleep disturbance in chronic back pain. The Clinical Journal of Pain. 1988;4(4):225-32.
7. Moldofsky H, Scarisbrick P. Induction of neurasthenic musculoskeletal pain syndrome by selective sleep stage deprivation. Psychosomatic medicine. 1976.
8. Wolfe F, Michaud K, Li T. Sleep disturbance in patients with rheumatoid arthritis: evaluation by medical outcomes study and visual analog sleep scales. The Journal of Rheumatology. 2006;33(10):1942-51.
9. Haack M, Mullington JM. Sustained sleep restriction reduces emotional and physical well-being. Pain. 2005;119(1-3):56-64.
10. Smith MT, Edwards RR, McCann UD, Haythornthwaite JA. The effects of sleep deprivation on pain inhibition and spontaneous pain in women. Sleep. 2007;30(4):494-505.
11. Kundermann B, Krieg J-C, Schreiber W, Lautenbacher S. The effects of sleep deprivation on pain. Pain Research and Management. 2004;9(1):25-32.
12. Lentz MJ, Landis CA, Rothermel J, Shaver JL. Effects of selective slow wave sleep disruption on musculoskeletal pain and fatigue in middle aged women. The Journal of rheumatology. 1999;26(7):1586-92.
13. Tang NK, Goodchild CE, Sanborn AN, Howard J, Salkovskis PM. Deciphering the temporal link between pain and sleep in a heterogeneous chronic pain patient sample: a multilevel daily process study. Sleep. 2012;35(5):675-87.
14. Cappuccio FP, Taggart FM, Kandala N-B, Currie A, Peile E, Stranges S, et al. Meta-analysis of short sleep duration and obesity in children and adults. Sleep. 2008;31(5):619-26.
15. Cappuccio FP, D'Elia L, Strazzullo P, Miller MA. Quantity and quality of sleep and incidence of type 2 diabetes: a systematic review and meta-analysis. Diabetes care. 2010;33(2):414-20.
16. Cappuccio FP, D'Elia L, Strazzullo P, Miller MA. Sleep duration and all-cause mortality: a systematic review and meta-analysis of prospective studies. Sleep. 2010;33(5):585-92.

For peer review only - http://bmjopen.bmj.com/site/about/guidelines.xhtml

17. Hoevenaar-Blom MP, Spijkerman AM, Kromhout D, van den Berg JF, Verschuren W. Sleep duration and sleep quality in relation to 12-year cardiovascular disease incidence: the MORGEN study. Sleep. 2011;34(11):1487-92.
18. Lou P, Chen P, Zhang L, Zhang P, Yu J, Zhang N, et al. Relation of sleep quality and sleep duration to type 2 diabetes: a population-based cross-sectional survey. BMJ open. 2012;2(4):e000956.
19. Ford DE, Kamerow DB. Epidemiologic study of sleep disturbances and psychiatric disorders: an opportunity for prevention? Jama. 1989;262(11):1479-84.
20. Patel SR, Blackwell T, Redline S, Ancoli-Israel S, Cauley JA, Hillier TA, et al. The association between sleep duration and obesity in older adults. International journal of obesity. 2008;32(12):1825.
21. Casarett D, Karlawish J, Sankar P, Hirschman K, Asch DA. Designing pain research from the patient's perspective: what trial end points are important to patients with chronic pain? Pain Medicine. 2001;2(4):309-16.
22. Turk DC, Dworkin RH, Revicki D, Harding G, Burke LB, Cella D, et al. Identifying important outcome domains for chronic pain clinical trials: an IMMPACT survey of people with pain. PAIN®. 2008;137(2):276-85.
23. Krystal AD. A compendium of placebo-controlled trials of the risks/benefits of pharmacological treatments for insomnia: the empirical basis for US clinical practice. Sleep medicine reviews. 2009;13(4):265-74.
24. de Gage SB, Bégaud B, Bazin F, Verdoux H, Dartigues J-F, Pérès K, et al. Benzodiazepine use and risk of dementia: prospective population based study. Bmj. 2012;345:e6231.
25. Glass J, Lanctôt KL, Herrmann N, Sproule BA, Busto UE. Sedative hypnotics in older people with insomnia: meta-analysis of risks and benefits. Bmj. 2005;331(7526):1169.
26. Kripke DF, Langer RD, Kline LE. Hypnotics' association with mortality or cancer: a matched cohort study. BMJ open. 2012;2(1):e000850.
27. Modén B, Merlo J, Ohlsson H, Rosvall M. Psychotropic drugs and falling accidents among the elderly: a nested case control study in the whole population of Scania, Sweden. Journal of Epidemiology & Community Health. 2010;64(5):440-6.
28. Stewart R, Besset A, Bebbington P, Brugha T, Lindesay J, Jenkins R, et al. Insomnia comorbidity and impact and hypnotic use by age group in a national survey population aged 16 to 74 years. Sleep. 2006;29(11):1391-7.
29. Jann M, Kennedy WK, Lopez G. Benzodiazepines: a major component in unintentional prescription drug overdoses with opioid analgesics. Journal of pharmacy practice. 2014;27(1):5-16.
30. Montgomery P, Dennis J. A systematic review of non-pharmacological therapies for sleep problems in later life. Sleep medicine reviews. 2004;8(1):47-62.
31. Morgenthaler T, Kramer M, Alessi C, Friedman L, Boehlecke B, Brown T, et al. Practice parameters for the psychological and behavioral treatment of insomnia: an update. An american academy of sleep medicine report. Sleep. 2006;29(11):1415.

For peer review only - http://bmjopen.bmj.com/site/about/guidelines.xhtml

32. MORIN C, CULBERT J, SCHWARTZ S. Nonpharmacological interventions for insomnia: a meta-analysis of treatment efficacy. The American journal of psychiatry. 1994;151(8):1172-80.
33. Sateia M, Doghramji K, Hauri P, Morin C. Evaluation of chronic insomnia. An American Academy of Sleep Medicine review. Sleep. 2000;23(2):243-308.
34. Trauer JM, Qian MY, Doyle JS, Rajaratnam SM, Cunnington D. Cognitive behavioral therapy for chronic insomnia: a systematic review and meta-analysis. Annals of internal medicine. 2015;163(3):191-204.
35. Tang NK, Lereya ST, Boulton H, Miller MA, Wolke D, Cappuccio FP. Nonpharmacological treatments of insomnia for long-term painful conditions: a systematic review and meta-analysis of patient-reported outcomes in randomized controlled trials. Sleep. 2015;38(11):1751-64.
36. Wilson S, Anderson K, Baldwin D, Dijk D-J, Espie A, Espie C, et al. British Association for Psychopharmacology consensus statement on evidence-based treatment of insomnia, parasomnias and circadian rhythm disorders: An update. Journal of Psychopharmacology. 2019:0269881119855343.
37. Tang NK. Cognitive-behavioral therapy for sleep abnormalities of chronic pain patients. Current rheumatology reports. 2009;11(6):451.
38. National Institute for Clinical Excellence. Guidance on the use of zaleplon, zolpidem and zopiclone for the short-term management of insomnia. 2004. Technology Appraisal Guidance.77.
39. Mack LJ, Rybarczyk BD. Behavioral treatment of insomnia: a proposal for a stepped-care approach to promote public health. Nature and science of sleep. 2011;3:87.
40. Perlis ML, Smith MT. How can we make CBT-I and other BSM services widely available? Journal of Clinical Sleep Medicine. 2008;4(01):11-3.
41. Gordon K, Rice H, Allcock N, Bell P, Dunbar M, Gilbert S, et al. Barriers to self-management of chronic pain in primary care: a qualitative focus group study. Br J Gen Pract. 2017;67(656):e209-e17.
42. Tang NK, Goodchild CE, Salkovskis PM. Hybrid cognitive-behaviour therapy for individuals with insomnia and chronic pain: a pilot randomised controlled trial. Behaviour research and therapy. 2012;50(12):814-21.
43. Berger AM, Kuhn BR, Farr LA, Von Essen SG, Chamberlain J, Lynch JC, et al. One-year outcomes of a behavioral therapy intervention trial on sleep quality and cancer-related fatigue. Journal of Clinical Oncology. 2009;27(35):6033.
44. Öst L-G, Ollendick TH. Brief, intensive and concentrated cognitive behavioral treatments for anxiety disorders in children: A systematic review and meta-analysis. Behaviour research and therapy. 2017;97:134-45.
45. Bevan A, Oldfield VB, Salkovskis PM. A qualitative study of the acceptability of an intensive format for the delivery of cognitive‐behavioural therapy for obsessive‐compulsive disorder. British Journal of Clinical Psychology. 2010;49(2):173-91.
46. Morgan K, Gregory P, Tomeny M, David BM, Gascoigne C. Self‐help treatment for insomnia symptoms associated with chronic conditions in older adults: A randomized controlled trial. Journal of the American Geriatrics Society. 2012;60(10):1803-10.

For peer review only - http://bmjopen.bmj.com/site/about/guidelines.xhtml

47. Espie CA, Inglis SJ, Tessier S, Harvey L. The clinical effectiveness of cognitive behaviour therapy for chronic insomnia: implementation and evaluation of a sleep clinic in general medical practice. Behaviour research and therapy. 2001;39(1):45-60.
48. Public Health England. National General Practice Profiles 2019 [Available from: https://fingertips.phe.org.uk/profile/general-practice.
49. Edinger JD, Bonnet MH, Bootzin RR, Doghramji K, Dorsey CM, Espie CA, et al. Derivation of Research Diagnostic Criteria for Insomnia: Report of an American Academy of Sleep Medicine Work Group. Sleep. 2004;27(8):1567-96.
50. American Psychiatric Association. Diagnostic and statistical manual of mental disorders 5th ed. Washington, D.C.: American Psychiatric Association; 2013.
51. Bastien CH, Vallières A, Morin CM. Validation of the Insomnia Severity Index as an outcome measure for insomnia research. Sleep medicine. 2001;2(4):297-307.
52. Cleeland CS, Chapman CR, Loeser JD. Measurement of pain by subjective report. In: Chapman CR, Loeser JD, editors. Advances in Pain Research and Therapy, Volume 12: Issues in Pain Measurement. New York: Raven Press; 1989. p. 391 - 403.
53. Smets E, Garssen B, Bonke Bd, De Haes J. The Multidimensional Fatigue Inventory (MFI) psychometric qualities of an instrument to assess fatigue. Journal of psychosomatic research. 1995;39(3):315-25.
54. Zigmond AS, Snaith RP. The hospital anxiety and depression scale. Acta psychiatrica scandinavica. 1983;67(6):361-70.
55. Group TE. EuroQol-a new facility for the measurement of health-related quality of life. Health policy. 1990;16(3):199-208.
56. Devlin NJ, Shah KK, Feng Y, Mulhern B, van Hout B. Valuing health ‐ related quality of life: An EQ ‐ 5 D ‐ 5 L value set for E ngland. Health economics. 2018;27(1):7-22.
57. Sullivan MJ, Bishop SR, Pivik J. The pain catastrophizing scale: development and validation. Psychological assessment. 1995;7(4):524.
58. Tang NK, Salkovskis PM, Hanna M. Mental defeat in chronic pain: initial exploration of the concept. The Clinical journal of pain. 2007;23(3):222-32.
59. Tang NK, Harvey AG. Correcting distorted perception of sleep in insomnia: a novel behavioural experiment? Behaviour research and therapy. 2004;42(1):27-39.
60. Morin CM, Vallières A, Ivers H. Dysfunctional beliefs and attitudes about sleep (DBAS): validation of a brief version (DBAS-16). Sleep. 2007;30(11):1547-54.
61. Afolalu EF, Moore C, Ramlee F, Goodchild CE, Tang NK. Development of the pain-related beliefs and attitudes about sleep (PBAS) scale for the assessment and treatment of insomnia comorbid with chronic pain. Journal of Clinical Sleep Medicine. 2016;12(09):1269-77.
62. Carney CE, Buysse DJ, Ancoli-Israel S, Edinger JD, Krystal AD, Lichstein KL, et al. The consensus sleep diary: standardizing prospective sleep self-monitoring. Sleep. 2012;35(2):287.
63. Braun V, Clarke V. Using thematic analysis in psychology. Qualitative research in psychology. 2006;3(2):77-101.

For peer review only - http://bmjopen.bmj.com/site/about/guidelines.xhtml

64. Johannes CB, Le TK, Zhou X, Johnston JA, Dworkin RH. The Prevalence of Chronic Pain in United States Adults: Results of an Internet-Based Survey. The Journal of Pain. 2010;11(11):1230-9.
65. Morin CM, Benca R. Chronic insomnia. The Lancet. 2012;379(9821):1129-41.
66. Morin CM, LeBlanc M, Daley M, Gregoire JP, Mérette C. Epidemiology of insomnia: Prevalence, self-help treatments, consultations, and determinants of help-seeking behaviors. Sleep Medicine. 2006;7(2):123-30.
67. van Hecke O, Torrance N, Smith BH. Chronic pain epidemiology and its clinical relevance. British Journal of Anaesthesia. 2013;111(1):13-8.
68. Stinson K, Tang NKY, Harvey AG. Barriers to treatment seeking in primary insomnia in the United Kingdom: a cross-sectional perspective. Sleep. 2006;29(12):1643-6.
69. Vitiello MV, McCurry SM, Shortreed SM, Balderson BH, Baker LD, Keefe FJ, et al. Cognitive - behavioral treatment for comorbid insomnia and osteoarthritis pain in primary care: The lifestyles randomized controlled trial. Journal of the American Geriatrics Society. 2013;61(6):947-56.
70. ISRCTN42499563. Health professional Administered Brief Insomnia Therapy (HABIT) trial 2019 [Available from: http://www.isrctn.com/ISRCTN42499563?q=&filters=&sort=&offset=2&totalResults=17076&page=1&pageSize=10&searchType=basic-search.
71. Blatch-Jones AJ, Pek W, Kirkpatrick E, Ashton-Key M. Role of feasibility and pilot studies in randomised controlled trials: a cross-sectional study. BMJ Open. 2018;8(9):e022233.
72. Thabane L, Ma J, Chu R, Cheng J, Ismaila A, Rios LP, et al. A tutorial on pilot studies: the what, why and how. BMC Medical Research Methodology. 2010;10(1):1.
73. Van Straten A, Cuijpers P. Self-help therapy for insomnia: a meta-analysis. Sleep medicine reviews. 2009;13(1):61-71.

For peer review only

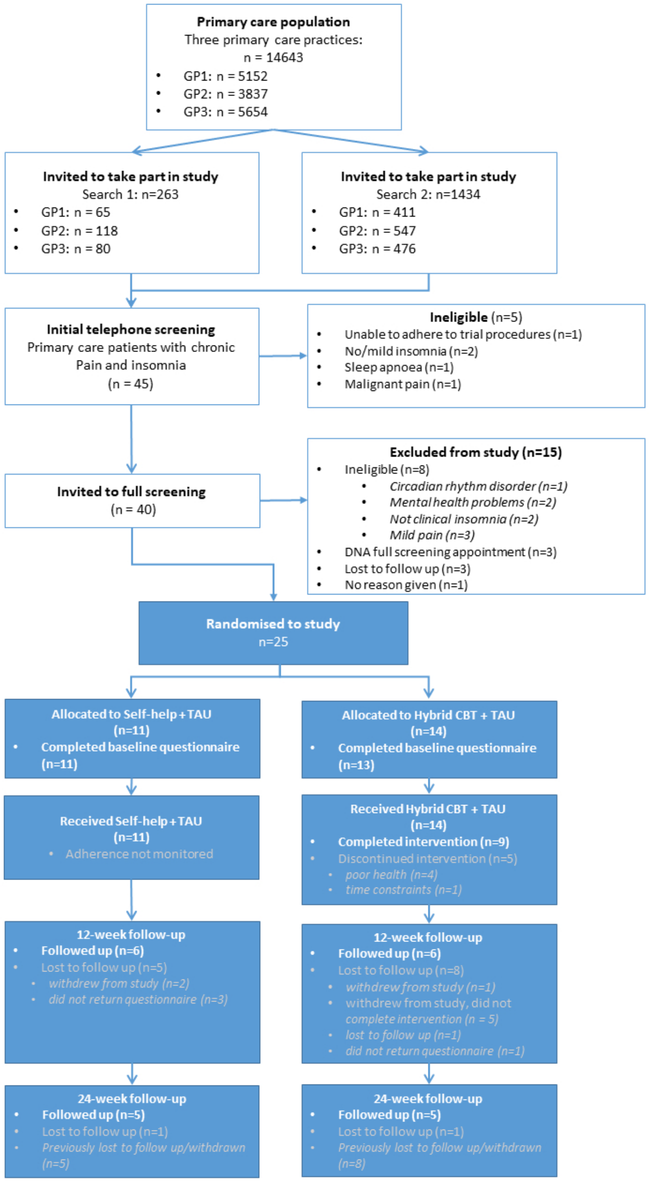

Figure 1. CONSORT flow diagram from screening (open boxes) to post-screening (filled boxes) processes in the study.

190x338mm (96 x 96 DPI)

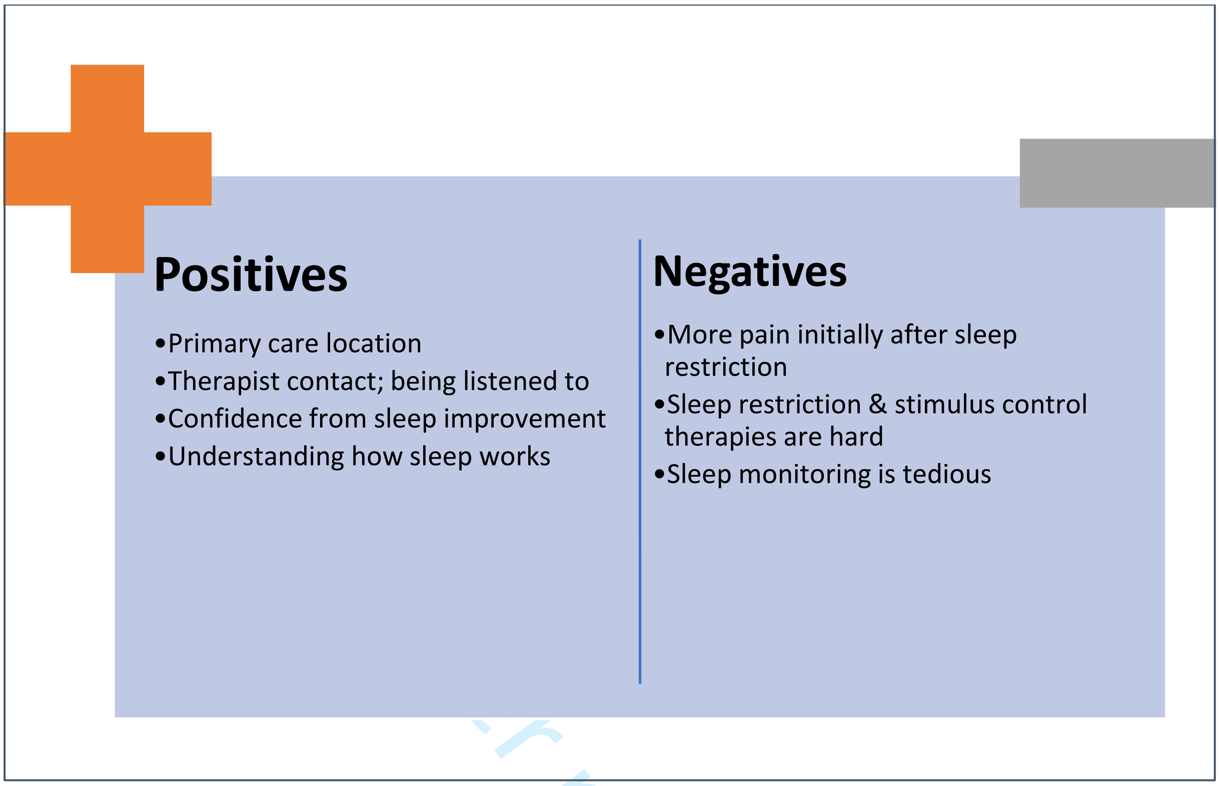

Figure 2. A summary of themes from post-intervention interviews of Hybrid CBT participants, highlighting the positives and negatives of the current treatment approach and content.

CONSORT 2010 checklist of information to include when reporting a pilot or feasibility trial*

Section/Topic	Item No	Checklist item	Reported on page No
**Title and abstract**			
	1a	Identification as a pilot or feasibility randomised trial in the title	1
	1b	Structured summary of pilot trial design, methods, results, and conclusions (for specific guidance see CONSORT abstract extension for pilot trials)	4-5
**Introduction**			
Background and objectives	2a	Scientific background and explanation of rationale for future definitive trial, and reasons for randomised pilot trial	6-7
	2b	Specific objectives or research questions for pilot trial	7
**Methods**			
Trial design	3a	Description of pilot trial design (such as parallel, factorial) including allocation ratio	8
	3b	Important changes to methods after pilot trial commencement (such as eligibility criteria), with reasons	n/a
Participants	4a	Eligibility criteria for participants	10-11
	4b	Settings and locations where the data were collected	8
	4c	How participants were identified and consented	10-11
Interventions	5	The interventions for each group with sufficient details to allow replication, including how and when they were actually administered	8-9
Outcomes	6a	Completely defined prespecified assessments or measurements to address each pilot trial objective specified in 2b, including how and when they were assessed	12-13, 13-14
	6b	Any changes to pilot trial assessments or measurements after the pilot trial commenced, with reasons	n/a
	6c	If applicable, prespecified criteria used to judge whether, or how, to proceed with future definitive trial	n/a
Sample size	7a	Rationale for numbers in the pilot trial	12
	7b	When applicable, explanation of any interim analyses and stopping guidelines	n/a
Randomisation:			
Sequence generation	8a	Method used to generate the random allocation sequence	11-12
	8b	Type of randomisation(s); details of any restriction (such as blocking and block size)	11-12
Allocation concealment mechanism	9	Mechanism used to implement the random allocation sequence (such as sequentially numbered containers), describing any steps taken to conceal the sequence until interventions were assigned	11-12

For peer review only

Implementation	10	Who generated the random allocation sequence, who enrolled participants, and who assigned participants to interventions	11-12
Blinding	11a	If done, who was blinded after assignment to interventions (for example, participants, care providers, those assessing outcomes) and how	11-12
	11b	If relevant, description of the similarity of interventions	9
Statistical methods	12	Methods used to address each pilot trial objective whether qualitative or quantitative	12-14
**Results**			
Participant flow (a diagram is strongly recommended)	13a	For each group, the numbers of participants who were approached and/or assessed for eligibility, randomly assigned, received intended treatment, and were assessed for each objective	14-15
	13b	For each group, losses and exclusions after randomisation, together with reasons	14-15
Recruitment	14a	Dates defining the periods of recruitment and follow-up	10, 12
	14b	Why the pilot trial ended or was stopped	n/a
Baseline data	15	A table showing baseline demographic and clinical characteristics for each group	Table 1
Numbers analysed	16	For each objective, number of participants (denominator) included in each analysis. If relevant, these numbers should be by randomised group	Table 2
Outcomes and estimation	17	For each objective, results including expressions of uncertainty (such as 95% confidence interval) for any estimates. If relevant, these results should be by randomised group	n/a
Ancillary analyses	18	Results of any other analyses performed that could be used to inform the future definitive trial	18-25
Harms	19	All important harms or unintended effects in each group (for specific guidance see CONSORT for harms)	
	19a	If relevant, other important unintended consequences	
**Discussion**			
Limitations	20	Pilot trial limitations, addressing sources of potential bias and remaining uncertainty about feasibility	28
Generalisability	21	Generalisability (applicability) of pilot trial methods and findings to future definitive trial and other studies	28
Interpretation	22	Interpretation consistent with pilot trial objectives and findings, balancing potential benefits and harms, and considering other relevant evidence	25-29
	22a	Implications for progression from pilot to future definitive trial, including any proposed amendments	25-29
**Other information**			
Registration	23	Registration number for pilot trial and name of trial registry	5, 8
Protocol	24	Where the pilot trial protocol can be accessed, if available	Suppl mat.
Funding	25	Sources of funding and other support (such as supply of drugs), role of funders	2-3
	26	Ethical approval or approval by research review committee, confirmed with reference number	3

For peer review only

Citation: Eldridge SM, Chan CL, Campbell MJ, Bond CM, Hopewell S, Thabane L, et al. CONSORT 2010 statement: extension to randomised pilot and feasibility trials. BMJ. 2016;355.
*We strongly recommend reading this statement in conjunction with the CONSORT 2010, extension to randomised pilot and feasibility trials, Explanation and Elaboration for important clarifications on all the items. If relevant, we also recommend reading CONSORT extensions for cluster randomised trials, non-inferiority and equivalence trials, non-pharmacological treatments, herbal interventions, and pragmatic trials. Additional extensions are forthcoming: for those and for up to date references relevant to this checklist, see www.consort-statement.org.

For peer review only

Consolidated criteria for reporting qualitative studies (COREQ): 32-item checklist

No	Item	Guide questions/description	Reported on Page No.
**Domain 1: Research team and reflexivity**			
	Personal Characteristics		
1.	Interviewer/facilitator	Which author/s conducted the interview or focus group?	13
2.	Credentials	What were the researcher's credentials? *E.g. PhD, MD*	1, 13-14
3.	Occupation	What was their occupation at the time of the study?	13-14
4.	Gender	Was the researcher male or female?	N/A (gender neutral language was used)
5.	Experience and training	What experience or training did the researcher have?	13-14
	Relationship with participants		
6.	Relationship established	Was a relationship established prior to study commencement?	13-14
7.	Participant knowledge of interviewer	What did the participants know about the researcher? *E.g. personal goals, reasons for doing the research.*	13-14
8.	Interviewer characteristics	What characteristics were reported about the interviewer/facilitator? *E.g. Bias, assumptions, reasons and interests in the research topic.*	13-14
**Domain 2: Study design**			
	Theoretical Framework		
9.	Methodological orientation and theory	What methodological orientation was stated to underpin the study? *E.g. grounded theory, discourse analysis, ethnography, phenomenology, content analysis*	14
	Participant Selection		
10.	Sampling	How were participants selected? *E.g. purposive, convenience, consecutive, snowball*	14-15, 18
11.	Method of approach	How were participants approached? *E.g. face-to-face, telephone, mail, email*	14-15, 18
12.	Sample size	How many participants were in the study?	14-15, 18
13.	Non-participation	How many people refused to participate or dropped out? Reason?	14-15, 18, Figure 1

For peer review only - http://bmjopen.bmj.com/site/about/guidelines.xhtml

Consolidated criteria for reporting qualitative studies (COREQ): 32-item checklist

	Setting		
14.	Setting of data collection	Where was the data collected? *E.g. home, clinic, workplace*	13-14
15.	Presence of non-participants	Was anyone else present besides the participants and researchers?	N/A
16.	Description of sample	What are the important characteristics of the sample? *E.g. demographic data and date*	13-14, 18 + Table 1, Table 3
	Data Collection		
17.	Interview guide	Were questions, prompts, guides provided by authors? Was it pilot tested?	13-14 + Box 1
18.	Repeat interviews	Were repeat interviews carried out? If yes, how many?	N/A; pre-post interview with different questions (13-14 + Box 1)
19.	Audio/visual recording	Did the research use audio or visual recording to collect the data?	14
20.	Field notes	Were field notes made during and/or after the interview or focus group?	14
21.	Duration	What was the duration of the interviews or focus group?	14
22.	Data saturation	Was data saturation discussed?	N/A
23.	Transcripts returned	Were transcripts returned to participants for comment and/or correction?	N/A
**Domain 3: Analysis and findings**			
	Data Analysis		
24.	Number of data coders	How many data coders coded the data?	13-14
25.	Description of the coding tree	Did authors provide a description of the coding tree?	N/A
26.	Derivation of themes	Were themes identified in advance or derived from the data?	14
27.	Software	What software, if applicable, was used to manage the data?	14
28.	Participant checking	Did participants provide feedback on the findings?	N/A
	Reporting		
29.	Quotations presented	Were participant quotations presented to illustrate the themes/findings? Was each quotation identified? *E.g. partipcipant number*	18-22; Table 3, Figure 2

For peer review only - http://bmjopen.bmj.com/site/about/guidelines.xhtml

Consolidated criteria for reporting qualitative studies (COREQ): 32-item checklist

30.	Data and findings consistent	Was there consistency between the data presented and the findings?	18-22; Table 3, Figure 2 + discussion
31.	Clarity of major themes	Were the major themes clearly presented in the findings?	Table 3 + Fig 2
32.	Clarity of minor themes	Is there a description of diverse cases or discussion of minor themes?	N/A

For peer review only
